# Modelling the structural variation of quartz and germanium dioxide with temperature by means of transformed crystallographic data

**DOI:** 10.1107/S2052520621002717

**Published:** 2021-05-20

**Authors:** Maximilian Fricke, Noel W. Thomas

**Affiliations:** a Werkstofftechnik Glas and Keramik, Hochschule Koblenz, Rheinstrasse 56, 56203 Hoehr-Grenzhausen, Germany

**Keywords:** quartz, pseudocubic, INA, tilt angle, structure prediction, GeO_2_

## Abstract

The pseudocubic (PC) parameterization of O_4_ tetrahedra [Reifenberg & Thomas (2018 ▸). *Acta Cryst.* B**74**, 165–181] is applied to quartz (SiO_2_) and its structural analogue germanium dioxide (GeO_2_). In α-quartz and GeO_2_, the pseudocubes are defined by three length parameters, *a*_PC_, *b*_PC_ and *c*_PC_, together with an angle parameter α_PC_. In β-quartz, α_PC_ has a fixed value of 90°. For quartz, the temperature evolution of parameters for the pseudocubes and the silicon ion network is established by reference to the structural refinements of Antao [*Acta Cryst.* (2016), B**72**, 249–262]. In α-quartz, the curve-fitting employed to express the non-linear temperature dependence of pseudocubic length and Si parameters exploits the model of a first-order Landau phase transition utilized by Grimm & Dorner [*J. Phys. Chem. Solids* (1975) ▸, **36**, 407–413]. Since values of tetrahedral tilt angles about 〈100〉 axes also result from the pseudocubic transformation, a curve for the observed non-monotonic variation of α_PC_ with temperature can also be fitted. Reverse transformation of curve-derived values of [Si+PC] parameters to crystallographic parameters *a*, *c*, *x*_Si_, *x*_O_, *y*_O_ and *z*_O_ at interpolated or extrapolated temperatures is demonstrated for α-quartz. A reverse transformation to crystallographic parameters *a*, *c*, *x*_O_ is likewise carried out for β-quartz. This capability corresponds to a method of structure prediction. Support for the applicability of the approach to GeO_2_ is provided by analysing the structural refinements of Haines *et al.* [*J. Solid State Chem.* (2002) ▸, **166**, 434–441]. An analysis of trends in tetrahedral distortion and tilt angle in α-quartz and GeO_2_ supports the view that GeO_2_ is a good model for quartz at high pressure.

## Abstract 2

A pseudocubic parameterization of the O_4_ tetrahedra of quartz and its structural analogue GeO_2_ at variable temperatures allows direct calculation of tetrahedral tilt angle, the microscopic order parameter of the α↔β phase transition. The crystal structures at interpolated or extrapolated temperatures can also be predicted.

## 1. Introduction   

Although the α↔β quartz inversion has been an issue of scientific investigation for some 130 years (Dolino, 1990 ▸), a strong stimulus to review current modelling methods for its crystal structures has been provided by the work of Antao (2016 ▸). By using synchrotron powder X-ray diffraction coupled with Rietveld structure refinements, she extended the range of structural data well into the temperature range of stability of β-quartz and provided a set of structural data for α- and β-quartz with a fine temperature mesh. A total of 67 new structural refinements resulted from her work, 42 for α-quartz and 25 for β-quartz, thereby providing an extensive dataset for structural analysis.

The foundation of several structural modelling studies of quartz and its homeotypes was laid by Grimm & Dorner (1975 ▸), who identified the tilt angle of δ of SiO_4_ tetrahedra about 〈100〉 axes in α-quartz as the microscopic order parameter in a first-order Landau model of the α↔β phase transition. Parameter δ_0_ in equation (1) corresponds to the jump in tilt angle at the transition temperature *T*_0_, with *T*_c_ a scaling parameter.
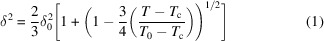
Grimm and Dorner (1975 ▸) assumed regular tetrahedra as a starting point for fitting equation (1) to values of δ derived from crystallographic data. This resulted in the values δ_0_ = 7.3°, *T*_0_ = 846 K and *T*_0_ − *T*_c_ = 10 K. They noted that the accuracy of the crystallographic data then available was insufficient to test the validity of equation (1), further that ‘a direct measurement of the tilt angle analogous to the case of SrTiO_3_ would be desirable.’

This notwithstanding, equation (1) has been widely adopted in subsequent studies of the temperature dependence of the structures of α-quartz and its homeotypes. This can be attributed to the greater suitability of δ or δ^2^ compared to direct temperature as an independent variable when describing the temperature dependence of structural parameters such as the Si—O—Si angle by means of low-order polynomials. It is appropriate, therefore, to regard δ, also denoted *Q* in later studies, as a temperature-derived tilt angle, irrespective of the degree of agreement with a structurally derived tilt angle.

Carpenter *et al.* (1998 ▸) adopted this approach to derive quadratic relationships between spontaneous strains *e*_1_ and *e*_3_ with *Q* ^2^. They also utilized the structural data of Kihara (1990 ▸) for α-quartz to reveal a linear relationship between the mean Si—O bond length and the square of the tilt angle. By virtue of her extensive structural dataset, Antao (2016 ▸) has further shown that strain parameters *e*_1_, *e*_3_, (*c*/*a*) and volume strain *V*_s_ vary linearly with *Q* ^2^ for α-quartz She also proposed linear relationships between atomic parameters *z*_O_ and *x*_Si_ with *Q*. Mean Si–Si distances and Si—O—Si angles were also shown to vary systematically with *Q*. In both cases, tilt angle was calculated according to the method of Grimm & Dorner (1975 ▸) assuming regular tetrahedra.

The structural refinements of Antao (2016 ▸) refer to space group *P*3_2_21 (No. 154) for α-quartz and space group *P*6_2_22 (No. 180) for β-quartz. The coordinates for α-quartz correspond to the *z*(+)-setting (Donnay & Le Page, 1978 ▸)^1^. When cooling right-handed β-quartz (space group *P*6_2_22), formation either of an α_1_ or an α_2_ trigonal structure depends on the sense of the tetrahedral tilting.^2^ These are in space groups *P*3_2_21 and *P*3_1_21, respectively. 

Ever since the early crystal-chemical treatments of quartz, the view has dominated that the SiO_4_ tetrahedra deviate insignificantly from perfect regularity. Megaw (1973*a* ▸) states this clearly: ‘We have already recognized the importance of a regular (or nearly regular) tetrahedron as a structure-building unit.’ In the seminal work of Grimm & Dorner (1975 ▸) in relating tetrahedral tilt angle to the Landau order parameter in equation (1), the assumption of regular tetrahedra was maintained as an expedient. Taylor (1984 ▸) explicitly called this assumption into question, to quote from his abstract: ‘Tilting models of framework compounds are critically examined and their failure to match the observed structural behaviour is attributed to changes in tetrahedral distortion. For quartz it appears that during compression the change in tetrahedral distortion is virtually all angular (O—Si—O angles), whereas during thermal expansion the change in distortion is in the Si—O distances. Such behaviour may typify the behaviour of many other framework compounds but the structural data needed to establish this are lacking.’

**Figure 1:**
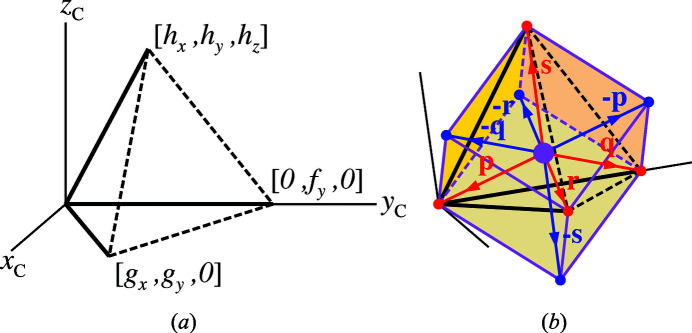
(*a*) The form of a generalized tetrahedron is defined by six parameters: *f_y_, g_x_, g_y_, h_x_, h_y_* and *h_z_*. These correspond to the non-zero components of its three bounding vectors in Cartesian coordinates. (*b*) A pseudocube is formed from the tetrahedron by inverting the four vectors from its centre-of-coordinates (large light purple circle) to oxygen ions, *i.e*. **p**, **q**, **r** and **s**, to form vectors −**p**, −**q**, −**r** and −**s**. Small red circles: pseudocube vertices occupied by oxygen ions; small blue circles: vacant pseudocube vertices (taken from Reifenberg & Thomas, 2018 ▸).

The current availability of high-quality structural data for quartz following the work of Antao (2016 ▸) now supersedes the final remark of Taylor for this framework compound. Furthermore, a new approach for quantifying the distortions of O_4_ tetrahedra has recently been proposed by Reifenberg & Thomas (2018 ▸). In the latter work, the pressure variation of the structure of the coesite polymorph of SiO_2_ was taken as a basis for defining a general procedure known as a pseudocubic transformation. Just as it is possible to generate a regular tetrahedron from a cube by taking two diagonally related corners of each cube face, the reverse procedure also holds: a regular tetrahedron will generate a regular cube, whereas a distorted tetrahedron will generate a distorted cube known as a pseudocube (Fig. 1 ▸). Such a pseudocube is, in general, characterized by six parameters, *a*_PC_, *b*_PC_, *c*_PC_, α_PC_, β_PC_ and γ_PC_ (Fig. 1 ▸), as for a triclinic unit cell. As shown in Fig. 1 ▸(*a*), the shape of a generalized tetrahedron is also defined by six parameters. It follows that all volumes and types of distortion of tetrahedral O_4_ cages can be quantitatively modelled by pseudocubic transformations.

Whereas the distorted O_4_ tetrahedra in coesite result in six independent pseudocubic parameters, the twofold symmetry axes through their centres-of-coordinates in α-quartz dictate that two of the pseudocubic angles are equal to 90°.

**Figure 2:**
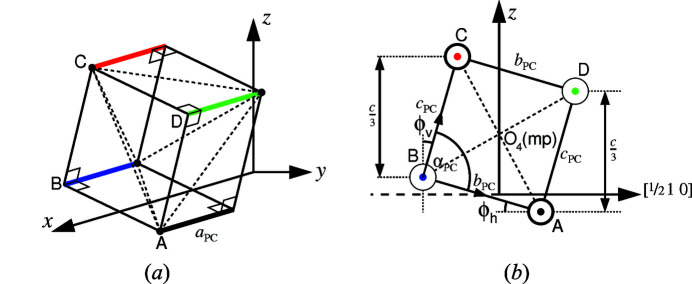
(*a*) The O_4_ tetrahedron corresponding to the silicon ion at *x*, 0, 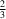 (as in space group *P*3_2_21) and its corresponding pseudocube. Length *a*_PC_ is shown. (*b*) The pseudocube shown in projection perpendicular to the *x*-axis [O_4_(mp): centre-of-coordinates of O_4_ tetrahedron and associated pseudocube; ϕ_h_,ϕ_v_: horizontally and vertically defined pseudocubic tilt angles]. (Modifed from an original figure given by Reifenberg & Thomas, 2018 ▸.)

This is shown in Fig. 2 ▸(*a*), in which pseudocubic axes *a*_PC_ are oriented parallel to the twofold axes. The face-on view of the pseudocube along the *x*-axis in Fig. 2 ▸(*b*) shows a parallelogram with twofold symmetry and internal angle α_PC_.

A secondary result of the pseudocubic transformation is that it allows angles ϕ_v_ and ϕ_h_ to be defined as direct indicators of tetrahedral tilt angle ϕ about the [100] axis, and more generally 〈100〉 axes: a tetrahedral rotation by this angle also leads to a rotation ϕ of its pseudocube about the same axis. However, unlike the tetrahedral edge vectors, the edge vectors of the pseudocube are aligned with the crystal axes. Angle ϕ_v_ is defined as the angle between pseudocubic axis *c*_PC_ and crystal axis *z* and angle ϕ_h_ as the angle between pseudocubic axis *b*_PC_ and its projection in the crystal *xy* plane. Owing to small deviations of pseudocubic angle α_PC_ from 90°, ϕ_h_ and ϕ_v_ are not exactly equal to each other. Nevertheless, a method is now provided for measuring the tetrahedral tilt angle directly, as sought by Grimm & Dorner (1975 ▸). The method does not require any approximations or abstract geometrical reference points other than the crystal axes. In the current work, the dependence of ϕ_h_, ϕ_v_ and mean tilt angle ϕ = (ϕ_h_ + ϕ_v_)/2 on temperature-derived tilt angle δ (or equivalently *Q*) are examined, thereby revealing the extent to which equation (1) holds for α-quartz.

**Figure 3:**
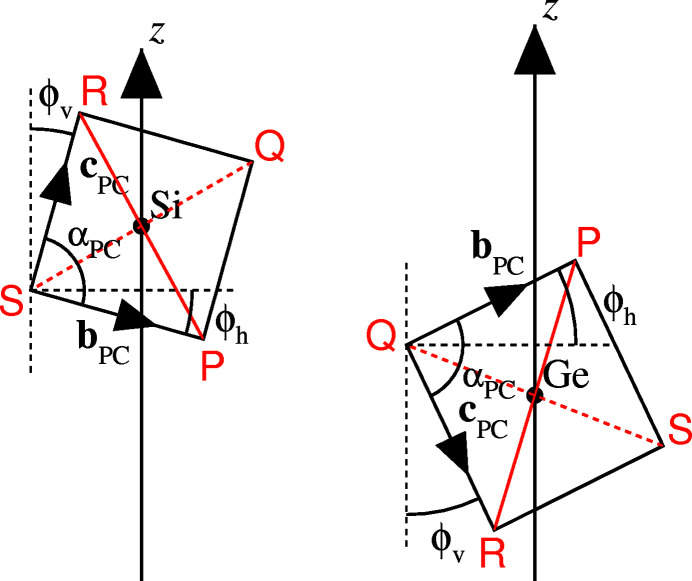
Oxygen ion pseudocubes (left) in α-quartz (space group *P*3_2_21) and (right) in GeO_2_ (space group *P*3_1_21) viewed along the crystallographic *x*-axis with the *z*-axis vertical. Vectors ***b***_PC_ and ***c***_PC_ are the two axes of the pseudocube that enclose angle α_PC_. Angles ϕ_h_ and ϕ_v_ are tilt angles with horizontal and vertical reference directions. Lines PR and QS are tetrahedral edges (solid lines: at front; dashed lines: at rear).

The significance of a *direct* measurement of tilt angle may be made clear by comparing the completely general pseudocubic method with alternative structural approaches advocated by Megaw (1973*b* ▸) and Grimm & Dorner (1975 ▸) for quartz, as well as the method of Haines *et al.* (2003 ▸) adopted for the quartz homeotype FePO_4_. Megaw adopted as a basis an idealized tetrahedron of orthorhombic symmetry, as in β-quartz, and maintained this form as an approximation in α-quartz. This approach is equivalent to allowing a pseudocube with unequal edge lengths but with angle α_PC_ fixed at 90°. The method of Grimm and Dorner is more restrictive, as it amounts to assuming a regular cube as the pseudocubic form. Haines *et al.*, by comparison, examined the deviations in orientation of tetrahedral edges PR and QS from ±45° (Fig. 3 ▸).

Fig. 3 ▸ shows the alternative senses of tilt in space group *P*3_2_21 for α-quartz and in its enantiomorphic space group *P*3_1_21, in which the 16 structures of GeO_2_ between 294 and 1344 K to be examined here were set (Haines *et al.*, 2002 ▸).^3^

**Figure 4:**
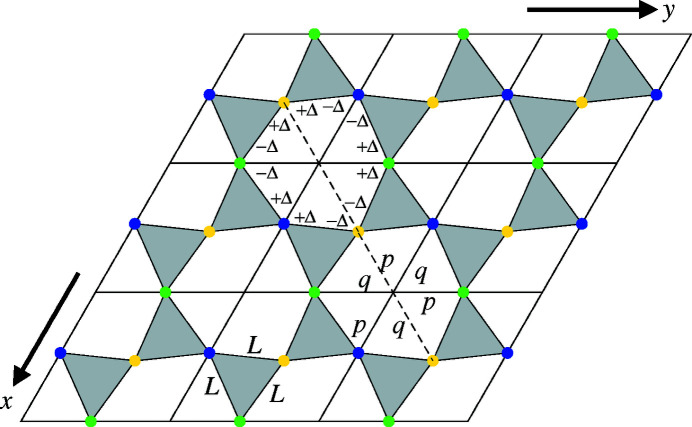
Silicon ions (blue, green and yellow circles) of α-quartz in *xy*-projection in space group *P*3_2_21 (blue: *z* = 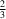; green: *z* = 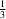; yellow: *z* = 0). These form a 2D framework characterized by two parameters, *L* (equilateral triangle side-length) and Δ (deviation of angle from 60° in constitutive triangle of hexagonal void). Lengths *p* and *q* show the unequal radii of the hexagonal voids, with *p* < *q* (taken from Figure 12 of Reifenberg and Thomas, 2018 ▸).

In addition to investigating the validity of equation (1) in describing the temperature variation of tilt angle as determined by the pseudocubic method, an important further aim of this work is to exploit the pseudocubic transformation for the purpose of structure prediction at temperatures outside the ranges of experimental investigation of Antao (2016 ▸) and Haines *et al.* (2002 ▸). Since the pseudocubes only relate to the oxygen ions, the silicon or germanium ions are treated in a separate cationic network. This is consistent with the general methodology of ionic network analysis (INA) (Thomas, 2017 ▸). In Fig. 4 ▸, the positions of the silicon ions along the screw axes in α-quartz have been collapsed on to the *xy* plane, in order to form a two-dimensional framework defined by parameters *L* and Δ. Δ is equal to zero in the higher-symmetry β-structure.

The crystal structures of α-quartz and GeO_2_ are defined by two unit-cell and four positional parameters, *i.e.* *a*, *c*, *x*_Si_, *x*_O_, *y*_O_ and *z*_O_, which are known collectively as six degrees of freedom (d.o.f.). In β-quartz, by comparison, there are three degrees of freedom^4^, *i.e.* *a*, *c* and *x*_O_. The question arises as to how many independent transformed parameters are required to define the O_4_ pseudocubes and silicon ion networks in the two quartz modifications. For α-quartz, six independent parameters are required, *i.e. a*_PC_, *b*_PC_, *c*_PC_, α_PC_, *L* and Δ. These match exactly the six d.o.f. of the structure. In β-quartz, just three independent parameters are required, although the pseudocubes and silicon ion network deliver four: *a*_PC_, *b*_PC_, *c*_PC_ and *L.* This disparity is resolved by noting that parameters *a*_PC_ and *b*_PC_ are interdependent.^5^ It should also be noted that the tetrahedral tilt angle in α-quartz, ϕ, is not a transformed parameter in this sense: if the six crystal structural parameters or alternatively the six independent transformed parameters are known, the value of ϕ follows by calculation.

This article is structured as follows. In §2, analytical expressions are given for the values of transformed parameters *a*_PC_, *b*_PC_, *c*_PC_, α_PC_, *L* and Δ, henceforth denoted [Si+PC] or [Ge+PC], in terms of crystal structural parameters *a*, *c*, *x*_Si_ or *x*_Ge_, *x*_O_, *y*_O_ and *z*_O_. An expression is also given for tilt angles ϕ_v_ and ϕ_h_ in terms of crystal structural parameters. In §3.1, the transformed parameters calculated for α-quartz are summarized by reference to Table S1 in §4 of the supporting information. Sections §3.2 to §3.4 refer to α-quartz: the temperature variation of the three tilt angles ϕ_v_ and ϕ_h_ and mean tilt angle ϕ = (ϕ_h_ + ϕ_v_)/2 is compared to the temperature-derived value of tilt angle according to equation (1) in §3.2. This equation is subsequently exploited as a baseline curve for a quantitative description of the variation of the three tilt angles with temperature. In §3.3, curves are derived for the temperature variation of [Si+PC] parameters in α-quartz, with their application for the purpose of structure prediction shown in §3.4. §3.5 deals with GeO_2_ as a whole, referring to Table S2 in §4 of the supporting information. In §3.6, β-quartz is likewise dealt with as a whole, with reference made to Table S3. In §4.1 a comparison of the temperature- and pressure-dependent behaviour of α-quartz and GeO_2_ is made, with a discussion of the significance of tetrahedral distortions in framework structures carried out in §4.2.

## 2. Parameterization of the cation frameworks and the O_4_ tetrahedra in α-quartz, GeO_2_ and β-quartz structures   

The analytical treatment here applies to the three space groups relevant to the experimental data of Antao (2016 ▸) and Haines *et al.* (2002 ▸), *i.e.* *P*3_2_21, *P*3_1_21 and *P*6_2_22. Although the notation *x*_Si_ is used, it is to be understood that this also applies to the *x*-coordinate for germanium in the GeO_2_ structure. The equations quoted here are derived as follows in the supporting information: §S1: cationic network parameters *L* and Δ in α-quartz and GeO_2_; §S2: PC parameters and tilt angles in α-quartz and GeO_2_; §S3: PC parameters in β-quartz. These derivations are based on the appropriate space group symmetry, in order to fix the Si or Ge ions in space and to form connected O_4_ tetrahedral cages.

### 2.1. The cationic network   

The transformations from *a* and *x*_Si_ to *L* and Δ for α-quartz are as follows:
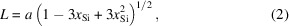


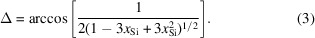
In the case of β-quartz, the value *x*_Si_ = 0.5 leads to the results *L* = *a*/2 and Δ = 0.

Reverse transformation from [Si+PC] to crystal structural parameters proceeds according to equations (4) and (5).
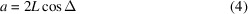
Quadratic equation (5) follows from equation (1):
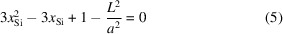
The smaller of the two roots corresponds to the value of *x*_Si_.

### 2.2. Pseudocubic parameters and tilt angles in α-quartz and germanium dioxide   

The six parameters of the pseudocubes for the O_4_ tetrahedra may be calculated as follows from unit-cell parameters *a* and *c* together with the *x*, *y* and *z* parameters of the oxygen ions: 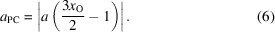
The expression for parameter *b*_PC_ depends on whether space group *P*3_2_21 or space group *P*3_1_21 applies, as for α-quartz and GeO_2_, respectively.
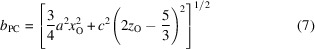
[for space group 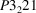], 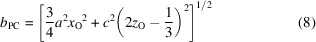
[for space group 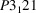], 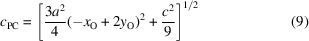
The results for parameter α_PC_ are likewise dependent on the space group that applies.
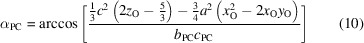
[for space group 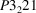] ,
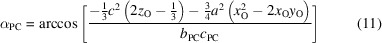
[for space group 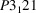]
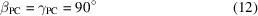
In Figs. 3 ▸(*a*) and 3 ▸(*b*), tilt angles ϕ_v_ and ϕ_h_ are shown for tetrahedra with cations at *x*_Si_, 0, 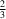 and *x*_Ge_, 0, 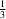 in α-quartz and GeO_2_, respectively. In both cases,
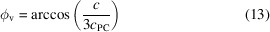
and
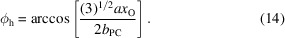
Calculation of the mean tilt-angle, 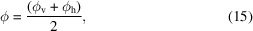
is straightforward. From the geometry in Fig. 3 ▸, it follows that
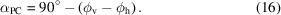
Equation (16) represents an alternative to equations (10) and (11) for calculating the pseudocubic angle α_PC_. It also reveals how deviations of the pseudocubic angle from 90° result from differences in the values of tilt-angles ϕ_v_ and ϕ_h_.

The INA method demands that reverse transformations from pseudocubic to crystal structural parameters can take place. In this connection, equations (4) and (5) relating to the cationic network enable this for cell parameter *a* and cation parameter *x*_Si_. The remaining four parameters, *i.e.* *c*, *x*_O_, *y*_O_, *z*_O_, may be calculated as follows from the pseudocubic parameters. Parameter *x*_O_ is derived from *a*_PC_ via equation (6). Parameters *c*, *y*_O_ and *z*_O_ are derived from the values of *b*_PC_, *c*_PC_ and α_PC_ by finding self-consistent solutions of equations (7) to (11) using numerical methods. These reverse transformations are carried out in §3.4 for α-quartz.

### 2.3. Pseudocubic parameters in β-quartz   

The six parameters of the pseudocubes for the O_4_ tetrahedra in β-quartz may similarly be calculated analytically from unit-cell parameters *a* and *c* together with the *x*_O_ parameter of the oxygen ions^6^:
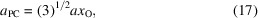


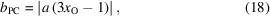


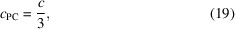


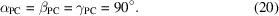
The lengths of pseudocubic axes *a*_PC_ and *b*_PC_ are interdependent, since both are determined by parameters *a* and *x*_O_. Parameter *L* in the cation network is equal to twice the unit-cell parameter *a*, and the pseudocubes yield values for *x*_0_ and *c* by reverse transformation. These transformations are carried out in §3.6.

## 3. The temperature variation of [Si+PC] parameters for quartz and [Ge+PC] parameters for germanium dioxide   

### 3.1. Parameters calculated for α-quartz   

[Si+PC] parameters calculated for α-quartz from the data of Antao (2016 ▸) are listed in Table S1. Also listed are the volumes of the unit cell (*V*_UC_), tetrahedral volumes (*V*_tetra_), the ratios of the volume occupied by tetrahedra to the unit-cell volume (3*V*_tetra_/*V*_UC_), the length-based tetrahedral distortion parameters (λ_PC_) [equation (21); Reifenberg & Thomas, 2018 ▸], together with tilt angles ϕ_v_ and ϕ_h_.
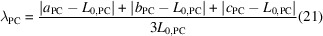
with *L*_0,PC_ = (*a*_PC_ + *b*_PC_ + *c*_PC_)/3.

### 3.2. Curve-fitting for the temperature variation of tilt angles in α-quartz   

**Figure 5:**
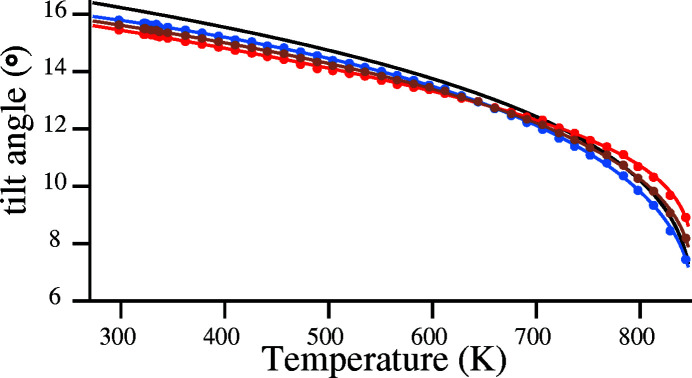
Comparison of the order parameter δ (black curve) with structurally derived values of tilt angles, ϕ_v_, ϕ_h_ and ϕ with points and curves in red, blue and brown, respectively. Temperature range: 273–846 K.

The correlation of values of order parameter δ calculated from equation (1) using the parameters of Grimm & Dorner (1975 ▸) with values of ϕ_v_, ϕ_h_ and ϕ calculated directly from the structural refinements of Antao (2016 ▸) via equations (13) to (15) is shown in Fig. 5 ▸.

It is observed that the correlation between the black curve and the other three curves is only qualitative. This indicates that, although the predominant contribution to the microscopic Landau order parameter is made by tetrahedral tilting, there will also be a small contribution to this from tetrahedral distortion.

Fitting of the curves linking experimental points for ϕ_v_, ϕ_h_ and ϕ_m_ was carried out by expressing these three parameters as a function of δ, using polynomials of order 3. The fitting coefficients are listed in Table 1 ▸.

### 3.3. Curve fitting for the temperature variation of [Si+PC]-parameters in α-quartz   

**Figure 6:**
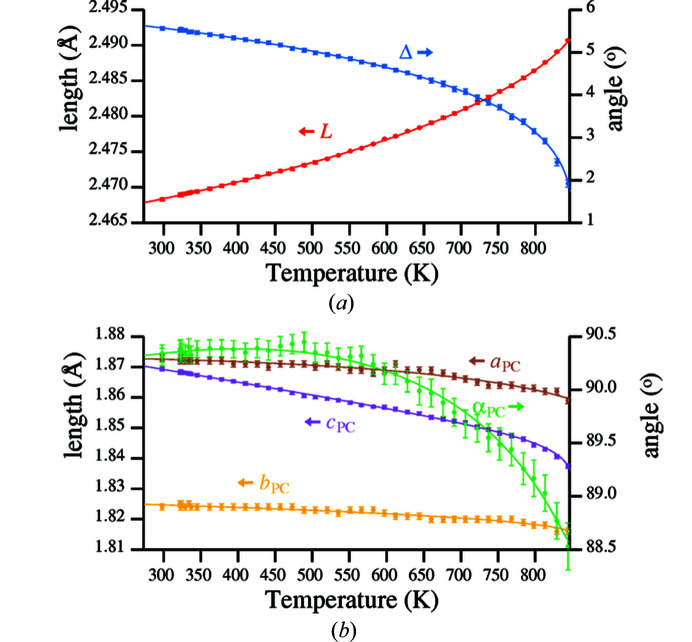
(*a*) Silicon framework parameters *L* and Δ; (*b*) oxygen pseudocube parameters *a*_PC_, *b*_PC_, *c*_PC_ and α_PC_.

Values of parameters *L*, Δ, *a*_PC_, *b*_PC_, *c*_PC_ and α_PC_ from Table S1 for temperatures between 298 and 844 K are plotted as points with associated error bars in Fig. 6 ▸.

The method adopted for fitting the curves was consistent with the work of other authors (Grimm & Dorner, 1975 ▸; Carpenter *et al.*, 1998 ▸; Antao, 2016 ▸), in that the order parameter δ generated by equation (1) was adopted as the independent variable. The fitting coefficients listed in Table 2 ▸ relate to the reduced order parameter δ′ defined in equation (22)
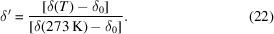
Here δ_0_ is the parameter of Grimm & Dorner (1975 ▸), which is equal to 7.3°. This is their tilt angle at the temperature *T*_0_, which is equal to 846 K. Parameter δ(273 K) is calculated by equation (1) to be 16.40°. δ(*T*) is the tilt angle calculated from equation (1) for a temperature lying between 273 and 846 K. Therefore equation (22) delivers a parameter between 0 and 1 for decreasing temperatures between 846 and 273 K, respectively. The fitted curves are shown in Fig. 6 ▸. It should be noted that the use of polynomial coefficients allows parameters *L*, Δ, *a*_PC_, *b*_PC_ and *c*_PC_ to vary independently of one another, even though a single Landau order parameter calculated from temperature according to equation (1) is at the core of the fitting method. As a formal contribution to the method, the Landau function provides a more linear baseline that enables the fitting of low-order polynomials. If the five parameter values were fitted directly to reduced temperature, a higher order would be required in order to accommodate the significant non-linearity in the parameter–variation in the region of the phase transition, *i.e.* at *T* ≤ *T*_*c*_. However, such a step would also introduce undesirable short-range artefacts in the fitted curves of questionable physical basis.

The curve fitted for parameter α_PC_ was calculated from equation (17) utilizing values for tilt angles ϕ_v_ and ϕ_h_ calculated from the coefficients in Table 1 ▸ and shown in Fig. 5 ▸.

### 3.4. Structural prediction for α-quartz via the INA method   

The fine temperature-mesh adopted by Antao (2016 ▸) means that there is more to be gained by calculating crystal structures outside the range of 298–844 K than by calculating structures at intermediate temperatures. Therefore four of the temperatures chosen for Table 3 ▸, 273 K, 283 K, 293 K and 846 K lie outside this range. A large separation in temperatures of 100 K has been chosen for temperatures within the range given by Antao (2016 ▸). Table 3 ▸ should be read from the top downwards. The first step is to calculate the Grimm and Dorner order parameter, δ, via equation (1). Thereafter parameters ϕ_v_ and ϕ_h_ are calculated via equation (*A*1.1)[App appa] and the fitting coefficients of Table 1 ▸. In Table 3 ▸, the equations used to calculate [Si+PC]-parameters from α_PC_ down to *c*_PC_ are listed in the right-hand column. Thereafter the equations used to calculate the crystallographic parameters by reverse transformation from [Si+PC]-parameters are quoted in this column.

Calculated crystal structural parameters at the ten temperatures chosen are quoted below the horizontal rule in Table 3 ▸. The final three parameters, *y*_O_, *z*_O_ and *c*, were calculated via an iterative process using the GRG algorithm within the Microsoft *Excel Solver* software environment. Self-consistent solutions to equations (7), (9) and (10) were sought, using trial values for these three parameters. Their values were refined in order to bring values of *b*_PC_, *c*_PC_ and α_PC_ calculated from these equations into agreement with the values calculated from the coefficients relating to equation (*A*1.2) and quoted in Table 3 ▸. An indication of the self-consistency of the method is provided by the values of r.m.s. deviation quoted in the final line of Table 3 ▸. This parameter is defined in equation (23).
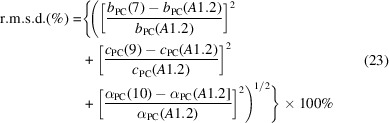
The numbers in the smallest brackets in equation (23) are equation numbers.

### 3.5. Tilt angles and [Ge+PC]-parameters for GeO_2_   

Although the 16 structures of GeO_2_ refer to temperatures between 294 and 1344 K (Haines *et al.*, 2002 ▸), the α-quartz-type structure for GeO_2_ is metastable with respect to a rutile-type phase at temperatures up to ∼1273 K. It is the equilibrium phase only at higher temperatures up to the melting point of ∼1390 K (Liu & Bassett, 1986 ▸). Landau parameters *T*_c_ and *T*_0_ as for α-quartz cannot be derived from structural data, as melting takes place on rising temperature before any such α→β phase transition.

Length-based parameters *L, a*_PC_, *b*_PC_, *c*_PC_ are larger for GeO_2_ than for α-quartz. Values of pseudocubic angle α_PC_ are also uniformly larger, lying in the range 91.11° ≤ α_PC_ ≤ 91.60°, compared to 88.53° ≤ α_PC_ ≤ 90.45° for α-quartz. This observation signifies a greater degree of angular distortion of the tetrahedra. Larger values of λ_PC_ also point to tetrahedra that are comparatively more distorted, as discussed further in §4.1. Values of the parameter 3*V*_tetra_/*V*_UC_ are higher for GeO_2_, this implying larger tilt angles: the greater the degree of tetrahedral tilting, the larger the proportion of space occupied by the tetrahedra. Tilt angles ϕ_v_ and ϕ_h_ are indeed consistently larger than for α-quartz, although they span narrower ranges: 22.36° ≤ ϕ_v_ ≤ 25.46°; 23.73° ≤ ϕ_h_ ≤ 26.63°. As for α-quartz, the smallest values in each range apply to the highest temperature. The implication is that GeO_2_ at 1344 K is still far away from an α→β phase transition.

The ability to measure tilt angles directly in this work was exploited by adopting mean tilt angle as the order parameter δ for GeO_2_ instead of an equation of the form of (1). A quadratic function was fitted to the experimental data for this purpose, as summarized in equation (24).
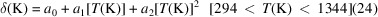
The following coefficients and r.m.s. deviation apply: *a*_0_ = 2.6242 × 10^1^; *a*_1_ = −4.0000 × 10^−4^; *a*_2_ = −1.5343 × 10^−6^; r.m.s.d.: 0.14°. Just as the thermal Landau order parameter allowed lower-order polynomials to be fitted for α-quartz, using the mean tilt angle here fulfils a similar purpose for the GeO_2_ fitting.

**Figure 7:**
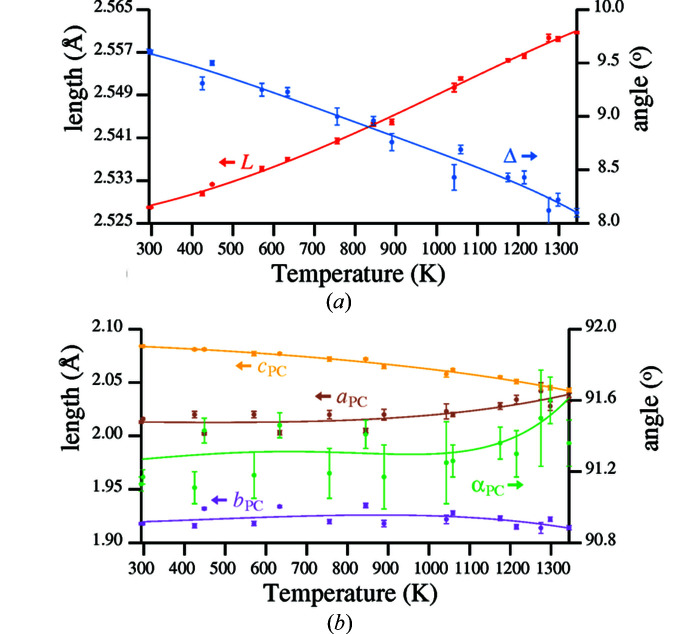
(*a*) Germanium framework parameters *L* and Δ; (*b*) oxygen pseudocube parameters *a*_PC_, *b*_PC_, *c*_PC_ and α_PC_.

Values of parameters *L*, Δ, *a*_PC_, *b*_PC_, *c*_PC_ and α_PC_ from Table S2 for temperatures between 294 and 1344 K are plotted as points with associated error bars in Fig. 7 ▸.

For the curve-fitting in Fig. 7 ▸, the order parameter δ generated by equation (24) was adopted as the independent variable. The fitting coefficients listed in Table 4 ▸ relate to the reduced order parameter δ′ defined in equation (25).
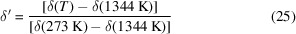
Thus δ′ = 0 at 1344 K and δ′ = 1 at 294 K. The curve fitted for parameter α_PC_ was calculated from equation (17), utilizing values for tilt angles ϕ_v_ and ϕ_h_ calculated from the coefficients in Table 5 ▸, using equation (*A*1.1).

Whereas the curves for *L*, Δ and *c*_PC_ lie mostly within the bounds of the error bars of the experimental points, this does not apply to parameters *a*_PC_, *b*_PC_ and α_PC_. It is further observed that successive experimental points for parameters *a*_PC_ and *b*_PC_ lie alternately above and below the fitted curves. At a given temperature, a point lying above the *a*_PC_ trend-curve corresponds to a point lying below the *b*_PC_ trend-curve, and *vice versa*. It transpires that points lying above the *a*_PC_ curve correspond to crystallographic data obtained from a sample measured with the Special Environment Powder Diffractometer at Argonne National Laboratory, whereas points lying below the curve relate to a different sample from the Polaris medium resolution diffractometer at the Rutherford Appleton Laboratory (Haines *et al.*, 2002 ▸). In both cases, the Rietveld method was used in conjunction with time-of-flight neutron powder diffraction data.

In view of the uncertainties in the values for parameters *a*_PC_, *b*_PC_ and α_PC_, it was decided not to proceed with calculations of crystallographic parameters at interpolated temperatures, as carried out in Table 3 ▸ for α-quartz. However, the separation of values for *a*_PC_, *b*_PC_ and *c*_PC_ into distinctive value-ranges is beyond question, this allowing a subsequent treatment of length-based tetrahedral distortion in §4. Owing to the systematic variation with temperature of INA parameters *L*, Δ and *c*_PC_, it is reasonable to assume that the INA method is applicable, in principle, to GeO_2_ over the whole temperature range. The observed fluctuations in the other parameters correlate with two different samples and experimental stations.

### 3.6. Curve-fitting and structural prediction for β-quartz   

**Figure 8:**
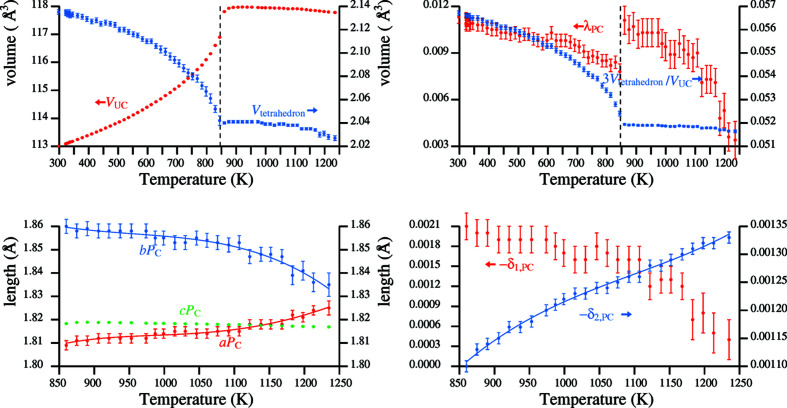
Parameter values for quartz derived from the data of Antao (2016 ▸): *top* over the whole temperature-range, *bottom* over the temperature range of β-quartz. *V*_UC_: unit-cell volume; *V*_tetrahedron_: SiO_4_-volume; λ_PC_: length-based tetrahedral distortion; *a*_PC_, *b*_PC_, *c*_PC_: pseudocubic parameters; δ_1,PC_, δ_2,PC_.

The evolution with temperature of several derived parameters for α- and β-quartz is shown in Fig. 8 ▸, based on the structural refinements of Antao (2016 ▸). The unit-cell volume increases uniformly with temperature in the α-phase and continues to rise beyond the phase transition to the β-phase to a maximum value at 921 K, before falling back gently with increasing temperature (Antao, 2016 ▸). The volumes occupied by the SiO_4_ tetrahedra decrease strongly with temperature in the α-phase, this being allowed by the decreasing mean tilt angle, and continue to fall gradually in the β-phase. The length-based tetrahedral distortion, λ_PC_, decreases with temperature in both phases, with a jump in values observed at the phase transition. Values ultimately attained at high temperature in the β-phase are lower than in the α-phase. Parameter 3*V*_tetrahedron_/*V*_UC_, which represents the fraction of space occupied by the SiO_4_ tetrahedra, decreases much more strongly in the α- than in the β-phase. In the former case, the decrease is facilitated by the reduction in mean tilt angle. In the latter, the decrease indicates the stronger relative decrease in tetrahedral volume compared to unit-cell volume.

Pseudocubic parameters *a*_PC_ and *b*_PC_ for β-quartz show a stronger temperature-dependence than *c*_PC_, with opposite trends observed for *a*_PC_ and *b*_PC_. Curves have been fitted to the variations for *a*_PC_ and *b*_PC_, since equations (17) and (18) yield, by reverse transformation, values of the *a* cell parameter and the oxygen *x*_O_ parameter.

The two parameters δ_1,PC_ and δ_2,PC_ are independent indicators of the deviation from regularity of the tetrahedra in β-quartz. They are defined as follows, whereby *x*_C_ is a reference value equal to 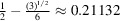 (see §S3.2 of the supporting information).
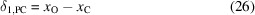


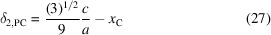
A perfectly regular tetrahedron would have both δ_1,PC_ and δ_2,PC_ equal to zero. The contrary motion of their negative values with increasing temperature in the fourth diagram of Fig. 8 ▸ indicates that perfect tetrahedral regularity is not attained in β-quartz.

The strong monotonic variation of − δ_2, PC_ with temperature allows a curve-fitting from which values of unit-cell parameter *c* can be derived. Taken together, parameters *a*_PC_, *b*_PC_ and δ_2, PC_ with associated curves enable prediction of the structures of β-quartz at interpolated temperatures. This procedure is shown in Table 6 ▸ for temperatures between 900 and 1200 K in 100 K intervals. The calculation procedure, which uses the coefficients listed in Table 7 ▸, may be inferred by reading the table from the top downwards.

## 4. Discussion   

### 4.1. Comparison of the temperature- and pressure-evolution of quartz and GeO_2_ structures by means of tetrahedral distortion parameters   

**Figure 9:**
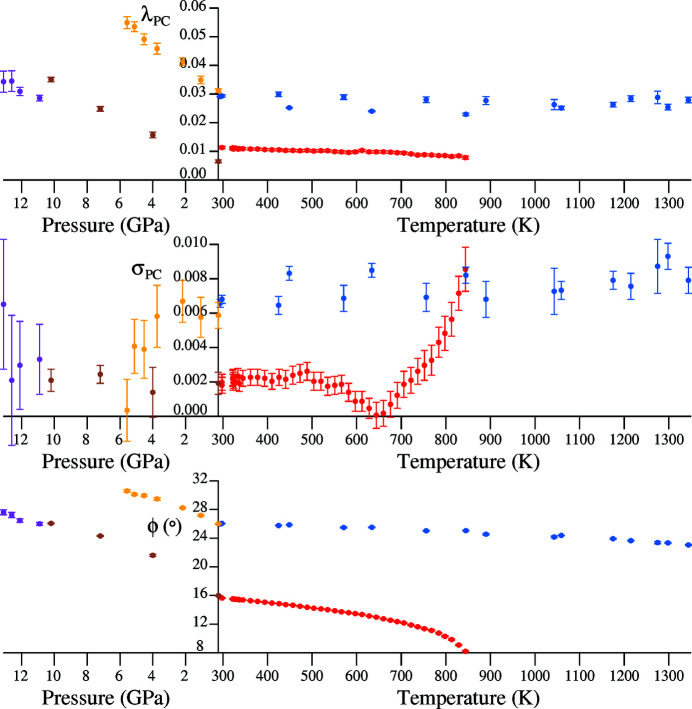
Comparison of tetrahedral distortions and tilt angles for α-quartz and GeO_2_. Red points: α-quartz at variable temperature (Antao, 2016 ▸); blue points: GeO_2_ at variable temperature (Haines *et al.*, 2002 ▸); brown points: pressure-variation of α-quartz up to 10.2 GPa (Glinnemann *et al.*, 1992 ▸) [The *r*(+) setting according to Donnay & Le Page (1978 ▸) in space group *P*3_1_21 was used. An origin-shift of 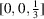 was applied in order to generate coordinates compatible with *International Tables for Crystallography* (Hahn, 1995 ▸)]; pink points: pressure variation of α-quartz between 10.9 and 13.1 GPa (Kim-Zajonz *et al.*, 1999 ▸) [The *r*(+) setting according to Donnay & Le Page (1978 ▸) in space group *P*3_1_21 was used with coordinates compatible with *International Tables for Crystallography* (Hahn, 1995 ▸).]; yellow points: pressure variation of GeO_2_ up to 5.57 GPa (Glinnemann *et al.* 1992 ▸).

The length- and angle-based tetrahedral distortion parameters, λ_PC_ and σ_PC_, introduced by Reifenberg & Thomas (2018 ▸) to enable a comparative overview of tetrahedral distortions under varying conditions of temperature and pressure, are plotted in Fig. 9 ▸ for α-quartz and GeO_2_. The former corresponds to equation (21) and the latter parameter 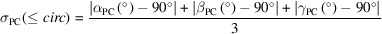
takes on the form of equation (28) when expressed in radians for α-quartz or GeO_2_. These two parameters correspond to normal and shear distortions, respectively, and are normalized in order to reflect changes in shape and not volume.
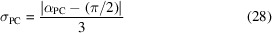
Also plotted are calculated values of mean tilt angle, ϕ, in degrees.

It is observed that λ_PC_ has uniformly higher values in GeO_2_ compared to α-quartz at a given temperature or pressure, and further, that the application of hydrostatic pressure increases the length-based distortion in both crystal structures. The behaviour of σ_PC_ is more complicated. The red points for α-quartz touch the *x*-axis at *circa* 640 K, when α_PC_ changes from values above 90° to values below 90° on increasing temperature. The blue points representing GeO_2_ are uniformly higher and show a weak dependence on temperature. By comparison, the application of pressures of up to 5.57 GPa to GeO_2_ causes σ_PC_ to fall off, corresponding to a reduction in α_PC_ from 91.0 to 89.9°. Such a fall-off is not observed for α-quartz, with a small upwards trend in σ_PC_ seen. This results from α_PC_ values that are consistently larger than 90°.

Although angular distortion σ_PC_ falls with increasing pressure in GeO_2_, this is not associated with the approach to a phase transition, as tilt angle ϕ takes on successively higher values with increasing pressure. This is the primary structural response of both GeO_2_ and α-quartz to increasing pressure.

Taken together, these results support the view expressed by Glinnemann *et al.* (1992 ▸) that unpressurized GeO_2_ is a good model of the high-pressure structure of α-quartz: the blue points for unpressurized GeO_2_ and the pink points for α-quartz at high pressure occupy similar regions along the *y*-axis in the three diagrams of Fig. 9 ▸.

### 4.2. The significance of tetrahedral distortion in quartz   

The term *distortion* implies deviation from an ideal. Two fundamental approaches are available for specifying such an ideal, the first referring to symmetry and the second to structure. The former leads naturally to considerations of group theory and the latter to crystal chemistry. In the case of quartz, as examined here, the aristotype corresponds to space group *P*6_2_22 for β-quartz. On cooling below 846 K, a displacive phase transition to its maximal sub-group *P*3_2_21 takes place, this corresponding to α-quartz. Bärnighausen (1980 ▸) has described this transition as lattice-equivalent (*translations­gleich*). The β→α transition involves the loss of the twofold rotation symmetry in the parent space group along 〈210〉 axes. It is therefore assigned the index 2 and notation *t2*.

In terms of structure, the dominant feature observed in the lower symmetry, trigonal phase is tetrahedral tilting around the remaining 〈100〉 twofold axes, along which the Si atoms lie. This twofold symmetry restricts the possible distortions of the SiO_4_ tetrahedra, such that the distortion of the O_4_ cages may be represented by pseudocubes in which two of the angles, β_PC_ and γ_PC_, are equal to 90° (Fig. 2 ▸). A corollary is that four independent parameters are required to describe this distortion. The term *pseudocube* also implies the existence of an ideal of higher symmetry, *i.e.* the *cube*, which would be specified completely by one parameter, *a*_PC_, since the following three constraints apply: (i) *b*_PC_ = *a*_PC_; (ii) *c*_PC_ = *a*_PC_; (iii) α_PC_ = 90°. Such a cube corresponds to a perfectly regular O_4_ tetrahedron.

Although space group symmetry allows regular SiO_4_ tetrahedra to exist in both β- and α-quartz, this ideal is not observed experimentally. For β-quartz, a regular O_4_ tetrahedron would impose restrictions on both oxygen parameter *x*_O_ and *c*/*a* ratio such that δ_1,PC_ = δ_2,PC_ = 0 [see equations (26) and (27) and the fourth diagram of Fig. 8 ▸]. For α-quartz, the possibility of the existence of perfectly regular tetrahedra has been addressed by Smith (1963 ▸), who showed that this would require the *c*/*a* ratio to be less than 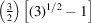. Equations (6), (7), (9) and (10) of the current work allow an extension of Smith’s analysis to examine the consequences of regular tetrahedra for tilt angle. The above three constraints to form a cube may be applied, together with a fourth constraint that the Si ion be located at the centre-of-coordinates of its O_4_-cage.

**Figure 10:**
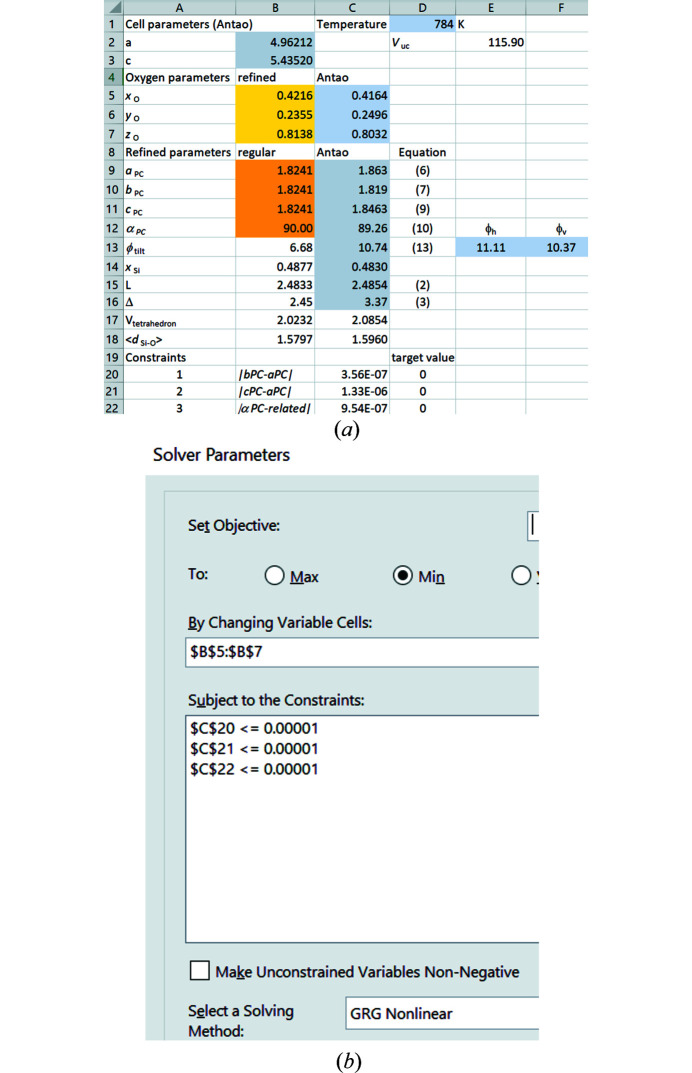
Procedure for calculating the oxygen positional parameters (*x*_O_,*y*_O_,*z*_O_) and silicon *x*-coordinate (*x*_Si_) of α-quartz with regular tetrahedra for fixed cell parameters *a* and *c*. (*a*) *EXCEL* spreadsheet. Cells B9 to B11 correspond to equations (6), (7) and (9), respectively. The formula in cell B12 calculates the pseudocubic angle *a*_PC_ according to equation (10). (*b*) Settings of the *Solver*.

Since Smith’s *c/a* criterion is fulfilled only by the nineteen structural refinements of Antao (2016 ▸) at temperatures *T* ≥ 566 K, one way to address this question is to take the values for *a* and *c* at these temperatures and to apply the four constraints in a Microsoft *Excel* spreadsheet supported by the iterative GRG refinement in the *Solver*. The spreadsheet used for an example structure at 784 K is shown in Fig. 10 ▸(*a*), with the *Solver* settings for constraints (i)–(iii) above shown in Fig. 10 ▸(*b*).

The values of cells B5–B7 are allowed to vary subject to the constraints that cells C20–C22 contain values less than 0.00001 at the end of the refinement. In this connection, cell C22 contains the difference of the two terms in the numerator of the argument to the arccos function in equation (10). This is zero for an α_PC_ angle of 90°. At the end of the refinement, cells B9–B12 (with light brown background) contain the parameters of a perfect cube. Further, the underlying equations, based on space group symmetry, guarantee that a system of interconnected regular SiO_4_ tetrahedra applies. The resulting oxygen *x*, *y*, *z* parameters necessary for this are given in cells B5–B7 (with yellow background). Significant differences are observed relative to the experimental parameters of Antao (2016 ▸) (cells C5–C7), to which irregular tetrahedra with pseudocubic parameters in cells C9–C12 apply. The value of *x*_Si_ in cell B14 is calculated by applying the fourth constraint relating to the location of the silicon ion at the centre-of-coordinates of its O_4_ cage. The associated values of *L* and Δ, which relate to the Si-ion framework, are quoted in cells B15 and B16 by application of equations (2) and (3). Equation (13) is used to calculate the tilt angle, ϕ_tilt_, resulting for the structure with regular tetrahedra. α_PC_ = 90° due to the regular tetrahedral geometry, so that ϕ_v_ = ϕ_h_. This is is quoted in cell B13, whereby the value of 6.68° is obtained for the *a* and *c* cell parameters of Antao (2016 ▸) at 784 K. This differs significantly from the experimental value of 10.74° (cell C13).

**Figure 11:**
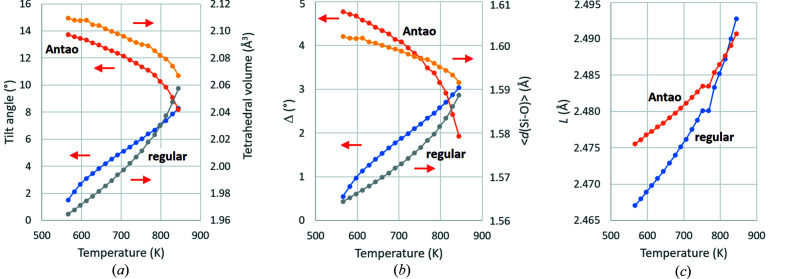
Comparison of structural variables for hypothetical α-quartz structures containing regular SiO_4_ tetrahedra with the experimental structures of Antao (2016 ▸) at temperatures between 566 and 844 K. (*a*) tilt angle ϕ and tetrahedral volume *V*_tetra_; (*b*) mean Si—O distance and Si-framework Δ-parameter; (*c*) Si-framework *L*-parameter.

It is significant that regular tetrahedra give rise to tilt angles that increase from 1.50 to 8.27° over the temperature range from 566 to 844 K, whereas the distorted tetrahedra in the experimental structures of Antao (2016 ▸) have tilt angles that decrease from 13.72 to 8.19° over this range [Fig. 11 ▸(*a*)]. That the primary Landau order parameter, *i.e.* tilt angle, should increase with increasing temperature is non-sensical. It follows that distorted tetrahedra in the α-phase are *necessary* for Landau theory to be applicable. This situation is at variance with the behaviour of perovskites, *i.e*. systems of interconnected *octahedra*. In this context, the group-theoretical analysis of Howard & Stokes (1998 ▸) found that, of the 15 possible sub-groups of cubic aristotype 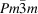 corresponding to different tilting patterns, only one was *necessarily* associated with octahedral distortion. They noted that such distortions were possible and expected in the other systems, but not required by geometry. These perovskite distortions have been analysed by other authors [see, for example, Thomas (1998 ▸); Tamazyan & van Smaalen (2007 ▸)].

Fig. 11 ▸(*a*) also demonstrates the expected correlation between tilt angle and tetrahedral volume for both regular and distorted tetrahedra. The larger tetrahedral volumes of distorted tetrahedra correlate with larger mean Si—O distances^7^,^8^ as well as angles Δ in the silicon ion framework [Fig. 11 ▸(*b*)]. The only case of parallel trends with temperature between regular and distorted tetrahedra relates to parameter *L* in the silicon ion framework [Fig. 11 ▸(*c*)]. In general, the distorted tetrahedra in the Antao structures permit relatively longer *L* values, leading to weaker Si⋯Si repulsions.

Violation of the criterion due to Smith (1963 ▸) does not allow a network of regular tetrahedra to be formed for the cell parameters obtained at temperatures below 566 K. His limiting *c*/*a*-ratio of 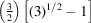 corresponds to a tilt angle of zero. However, equations (6) to (10) allow an interconnected network provided that one of the constraints encoded in cells C20 to C22 of Fig. 10 ▸(*a*) is relaxed. The results yielded by the Microsoft *Excel Solver* for a representative structure at 345 K are given in Table 8 ▸.

**Figure 12:**
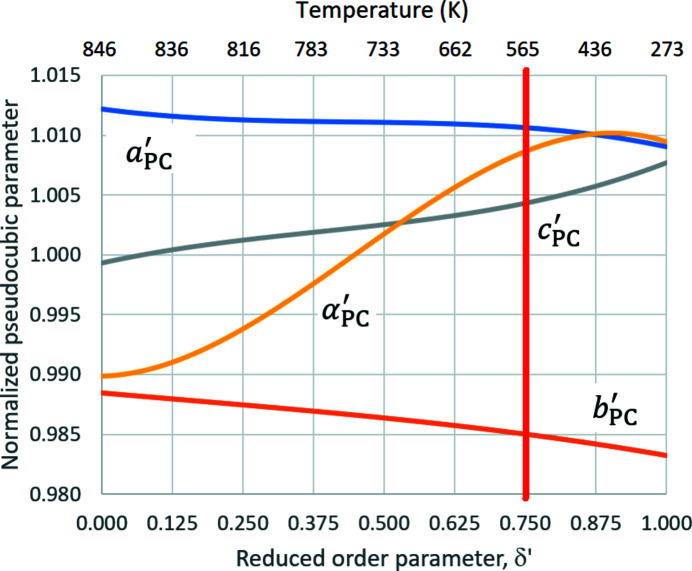
Variation of 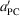, 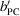, 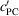 and 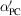 with reduced order parameter δ′ for α-quartz. Temperatures corresponding to values of δ′ are indicated at the top of the graph. Regular tetrahedra are only possible at values of δ′ up to *circa* 0.75, as denoted by the red vertical line.

The parameters obtained are strongly dependent on the constraints applied. Tilt angle ϕ is highly variable and is to be compared with the experimental mean tilt angle at this temperature of 15.36°. Given this sensitivity, a further issue is to examine the pseudocubic parameter combinations that apply to all the experimental structures of Antao (2016 ▸). To this end, the polynomials in Table 2 ▸ for these parameters are plotted as a function of reduced Landau order parameter δ′ [as defined in equation (22)] in Fig. 12 ▸. Since it is not possible to adopt a regular tetrahedron (or equivalently perfect cube) as a reference over the whole temperature, another method of normalization has been adopted: values of *a*_PC_, *b*_PC_ and *c*_PC_ have been divided by the mean of the three values at each temperature. In the case of α_PC_, absolute values have been divided by their median value (89.48°) over the whole temperature range. The corresponding normalized parameters, for which expansion/contraction effects have been factored out, are denoted by *a*_PC_′, *b*_PC_′, *c*_PC_′ and α_PC_′.

The modes of distortion of the O_4_ tetrahedra vary over the temperature range investigated, with the extent of the variation in parameters increasing in the order *a*_PC_′ < *b*_PC_′ < *c*_PC_′. The values of parameters *a*_PC_′ and *c*_PC_′ approach each another as δ′ → 1. This behaviour is close to the third pair of constraints in Table 8 ▸, for which the maximum tilt angle ϕ is observed. The unique increase in 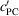 with increasing order parameter (→ tilt angle) may be rationalized by noting that increased tilt angles allow progressively larger values of *c*_PC_ to be accommodated for a given *c* cell parameter. This analysis also allows an independent assessment of the validity of the rigid unit (phonon) mode (RUM) approximation, according to which displacive phase transitions in framework structures occur without any significant distortion of the MO_4_ tetrahedra (O’Keeffe & Hyde, 1976 ▸; Giddy *et al.*, 1993 ▸).

The ability to generate structural models for α-quartz with alternative modes of tetrahedral distortion within the Microsoft *Excel Solver*, as shown in Fig. 10 ▸ and Table 8 ▸, is a useful by-product of the approach. Since this activity can be conducted independently of experimental diffraction data, it constitutes a simple, but versatile model-building method. It is likely to be useful for the modelling of auxetic (*i.e.* negative Poisson’s ratio) or non-auxetic behaviour of α-quartz subject to different constraints. Pioneering modelling work has been carried out here by Alderson & Evans (2009 ▸), in which alternative combinations of tetrahedral rotation and dilation were examined.

Since the [Si+PC] parameters can be reverse-transformed to crystallographic parameters, the method also allows the prediction of crystal structure at interpolated or extrapolated temperatures. This process, along with INA methods in general (Thomas, 2017 ▸; Reifenberg & Thomas, 2018 ▸) will be of benefit when carrying out structural refinements of lower symmetry structures. The use of alternative, group-theoretical methods in this context was pioneered by Stokes & Hatch (1988 ▸) and resulted in the *ISOTROPY* suite of programs (https://iso.byu.edu/iso/isotropy.php). In particular, the *ISODISTORT* web-based tool (Campbell *et al.*, 2006 ▸), which acts as a gateway to the *ISOTROPY* suite, is geared towards analysing structural distortions. This proceeds by identifying the irreducible representations of parent space groups that are associated with distortions in their sub-groups. It would therefore be worthwhile to attempt a synthesis of the two approaches towards the quartz phase transition, both crystal chemical and group-theoretical.

It is not surprising that length- and angle-based parameters vary smoothly with temperature and pressure, since they fundamentally reflect the interactional potential energies and vibrational energies of the ions. This observation underlies the importance of crystallographic experiments carried out under variable (*p*,*T*) conditions: they probe structural space. Furthermore, when lengths and angles are calculated, the complementary unit cell and atomic positional crystallographic parameters are combined in a Cartesian space that is conducive to establishing smooth trends with (*p*,*T*). This is the essential purpose of the transformation from crystallographic to [Si+PC] or, more generally, INA parameters. It therefore constitutes a technique that could become widely used in the refinement of structures examined under variable (*p*,*T*)-conditions.

It is intended to extend the current method to formulate more detailed structure-pieozelectric property relationships for single-crystal phosphates and arsenates (*ABO*_4_; *A* = B,Al,Ga,Fe; *B* = P,As) (Baumgartner *et al.*, 1984 ▸, 1989 ▸; Sowa, 1991 ▸, 1994 ▸; Nakae *et al.*, 1995 ▸; Haines *et al.*, 2004 ▸). These are homeotypic with α-quartz and GeO_2_. However, the presence of two different cations leads to two symmetry-independent tetrahedra in the unit cell. For this reason, their structures have not been analysed here. However, continued application of the tilted *regular* tetrahedron model to these materials (Krempl, 2005 ▸) points to a need to discriminate more clearly between tetrahedral tilt and distortion in these materials.

The additional insight regarding tetrahedral distortions in quartz made possible by the data of Antao (2016 ▸) signals how high-quality crystallographic data can also contribute to a deeper understanding of phase transitions. This should act as a spur towards the more regular collection of crystallographic data of superior quality.

In seeking a microscopic interpretation of the Landau order parameter, attention in the literature has been focused until now on the tilt angle of the tetrahedra. This is indeed the dominant contribution. However, the inability of regular tetrahedra to generate appropriate values of tilt angle, as found here, demonstrates the importance of also taking tetrahedral distortion explicitly into consideration. Thus the comment of Taylor (1984 ▸), that (purely) ‘tilting models of framework compounds fail to match the observed structural behaviour’, has been addressed.

In general, the potential of crystal chemistry is far greater than merely offering a descriptive post-rationalization of experimentally determined structures. It is also able to offer a predictive framework for detailed dialogue with experiment.

## Supplementary Material

The supporting information contains a derivation of the equations in Section 2 of the main article from first principles. DOI: 10.1107/S2052520621002717/ra5093sup1.pdf

## Figures and Tables

**Table 3:** [Si+PC]-parameters for α-quartz at ten temperatures calculated from the INA curves in Fig. 6 ▸ and associated polynomial coefficients in Tables 1 ▸ and 2 ▸ Corresponding, calculated crystallographic parameters are listed below the horizontal rule.

*T* (K)	273	283	293	300	400	500	600	700	800	846	Equation
δ (°)	16.404	16.342	16.279	16.235	15.549	14.744	13.753	12.427	10.191	7.300	(1)
ϕ_v_ (°)	15.607	15.548	15.488	15.446	14.817	14.121	13.316	12.307	10.706	8.612	(*A*1.1)
ϕ_h_ (°)	15.927	15.877	15.826	15.789	15.203	14.461	13.488	12.121	9.803	7.181	(*A*1.1)
α_PC_ (°)	90.320	90.329	90.338	90.343	90.386	90.340	90.172	89.814	89.097	88.569	(16)
δ′ (°)	1.0000	0.9932	0.9863	0.9814	0.9060	0.8176	0.7088	0.5631	0.3176	0.0000	(22)
*L* (Å)	2.46782	2.46804	2.46825	2.46841	2.47077	2.47350	2.47675	2.48083	2.48658	2.49095	(*A*1.2)
Δ (°)	5.62656	5.60521	5.58350	5.56808	5.32508	5.02677	4.64175	4.09670	3.11159	1.73590	(*A*1.2)
*a*_PC_ (Å)	1.87272	1.87266	1.87260	1.87255	1.87174	1.87057	1.86888	1.86640	1.86239	1.85951	(*A*1.2)
*b*_PC_ (Å)	1.82490	1.82482	1.82475	1.82469	1.82386	1.82291	1.82178	1.82036	1.81821	1.81594	(*A*1.2)
*c*_PC_ (Å)	1.87031	1.86990	1.86949	1.86920	1.86502	1.86076	1.85631	1.85142	1.84475	1.83589	(*A*1.2)
*a* (Å)	4.9119	4.9125	4.9131	4.9135	4.9202	4.9280	4.9373	4.9490	4.9658	4.9796	(4)
*x*_Si_	0.4716	0.4717	0.4718	0.4719	0.4731	0.4746	0.4766	0.4793	0.4843	0.4913	(5)
*x*_O_	0.4125	0.4125	0.4126	0.4126	0.4131	0.4136	0.4143	0.4152	0.4166	0.4177	(6)
*y*_O_	0.2655	0.2652	0.2650	0.2649	0.2625	0.2600	0.2572	0.2537	0.2481	0.2410	(7)
*z*_O_	0.7869	0.7871	0.7872	0.7874	0.7891	0.7913	0.7941	0.7981	0.8049	0.8123	(9)
*c* (Å)	5.4035	5.4039	5.4044	5.4046	5.4090	5.4137	5.4192	5.4265	5.4383	5.4444	(10)
r.m.s.d. (%)	0.0003	0.0002	0.0003	0.0003	0.0007	0.0002	0.0008	0.0003	0.0009	0.0011	

**Table 6:** Structural parameters of β-quartz at four temperatures calculated from the three fitted curves in Fig. 8 ▸ and associated polynomial coefficients in Table 7 ▸ †

*T* (K)	900	1000	1100	1200	Equation
δ′	0.1067	0.3733	0.6400	0.9067	(*A*2.1)
*a*_PC_ (Å)	1.8117	1.8135	1.8158	1.8220	(*A*1.2); Table 7 ▸
*b*_PC_ (Å)	1.8582	1.8558	1.8513	1.8399	(*A*1.2); Table 7 ▸
−δ_2,PC_ (Å)	0.001146	0.001215	0.001265	0.001314	(*A*1.2); Table 7 ▸
*a* (Å)	4.9961	4.9967	4.9963	4.9956	(17)
*x*	0.2094	0.2095	0.2098	0.2106	(18)
*c* (Å)	5.4563	5.4553	5.4535	5.4515	(19)

† Values of *x* are in keeping with *International Tables for Crystallography* (Hahn, 1995 ▸) and not the convention employed by Antao (2016 ▸).

**Table 8:** Pseudocubic parameters and tilt angles ϕ for the cell parameters of Antao (2016 ▸) at 345 K (*a* = 4.91637 Å; *c* = 5.40666 Å) that result from four alternative sets of two applied constraints

	Constraints applied			
Parameter	*b*_PC_ = *a*_PC_ {\alpha _{\rm PC}} = 90^\circ	*c*_PC_ = *b*_PC_ {\alpha _{\rm PC}} = 90^\circ	*a*_PC_ = *c*_PC_ {\alpha _{\rm PC}} = 90^\circ	*b*_PC_ = *a*_PC_ *c*_PC_ = *a*_PC_
*a*_PC_ (Å)	1.8308	1.7948	1.8558	1.8083
*b*_PC_ (Å)	1.8308	1.8474	1.8196	1.8083
*c*_PC_ (Å)	1.8522	1.8474	1.8558	1.8083
α_PC_ (°)	90.00	90.00	90.00	92.40
ϕ (°)	13.33	12.69	13.81	5.89

**Table 1:** Fitting coefficients for tilt angles ϕ_v_, ϕ_h_ and ϕ in α-quartz

	ϕ_v_ (°)	ϕ_h_ (°)	ϕ (°)
*a*_0_	1.19197 × 10^0^	6.32541 × 10^0^	3.77495 × 10^0^
*a*_1_	1.38790 × 10^0^	−7.60768 × 10^−1^	3.08692 × 10^−1^
*a*_2_	−6.68003 × 10^−2^	1.50911 × 10^−1^	4.21756 × 10^−2^
*a*_3_	2.18006 × 10^−3^	−4.19733 × 10^−3^	−1.00113 × 10^−3^
r.m.s.d. (°)	3.82 × 10^−2^	6.16 × 10^−8^	1.60 × 10^−8^

**Table 2:** Fitting coefficients for parameters *L*, Δ, *a*_PC_, *b*_PC_ and *c*_PC_ in α-quartz [equation (*A*1.2)]

Parameter	*a*_0_	*a*_1_	*a*_2_	*a*_3_	r.m.s.d.
*L* (Å)	2.49095 × 10^0^	−6.87965 × 10^−3^	−2.41458 × 10^−2^	7.89793 × 10^−3^	5.12 × 10^−5^
Δ (°)	1.73590 × 10^0^	4.47954 × 10^0^	−4.07902 × 10^−1^	−1.80983 × 10^−1^	2.14 × 10^−2^
*a*_PC_ (Å)	1.85951 × 10^0^	2.23242 × 10^−3^	2.64983 × 10^−2^	−1.55218 × 10^−2^	6.98 × 10^−4^
*b*_PC_ (Å)	1.81594 × 10^0^	6.10459 × 10^−3^	3.41215 × 10^−3^	−5.56681 × 10^−4^	4.19 × 10^−4^
*c*_PC_ (Å)	1.83589 × 10^0^	3.27551 × 10^−2^	−2.31572 × 10^−2^	2.48268 × 10^−2^	2.99 × 10^−4^

**Table 4:** Fitting coefficients for parameters *L*, Δ, *a*_PC_, *b*_PC_ and *c*_PC_ in GeO_2_ [equation (*A*1.2)]

Parameter	*a*_0_	*a*_1_	*a*_2_	*a*_3_	r.m.s.d.
*L* (Å)	2.56107 × 10^0^	−2.07438 × 10^−2^	−1.42516 × 10^−2^	2.19897 × 10^−3^	5.22 × 10^−4^
Δ (°)	8.09667 × 10^0^	1.44548 × 10^0^	−7.63365 × 10^−1^	8.11500 × 10^−1^	7.98 × 10^−2^
*a*_PC_ (Å)	2.03903 × 10^0^	−5.80389 × 10^−2^	3.20194 × 10^−2^	–	6.33 × 10^−3^
*b*_PC_ (Å)	1.91359 × 10^0^	4.23398 × 10^−2^	−3.62357 × 10^−2^	–	5.82 × 10^−3^
*c*_PC_ (Å)	2.04208 × 10^0^	5.27147 × 10^−2^	−1.94495 × 10^−2^	8.41710 × 10^−3^	1.28 × 10^−3^

**Table 5:** Fitting coefficients for tilt angles ϕ_v_ and ϕ_h_ in GeO_2_

	ϕ_v_ (°)	ϕ_h_ (°)
*a*_0_	−9.3270 × 10^2^	−1.7978 × 10^2^
*a*_1_	1.1504 × 10^2^	2.4388 × 10^1^
*a*_2_	−4.6455 × 10^0^	−1.0021 × 10^0^
*a*_3_	6.3008 × 10^−2^	1.4212 × 10^−2^
r.m.s.d. (°)	7.54 × 10^−2^	1.44 × 10^−1^

**Table 7:** Fitting coefficients for parameters *a*_PC_, *b*_PC_ and −δ_2,PC_ in β-quartz

Parameter	*a*_0_	*a*_1_	*a*_2_	*a*_3_	*a*_4_	r.m.s.d.
*a*_PC_ (Å)	1.8099 × 10^0^	2.1919 × 10^−2^	−5.6548 × 10^−2^	6.9719 × 10^−2^	−1.9627 × 10^−2^	8.19 × 10^−4^
*b*_PC_ (Å)	1.8596 × 10^0^	−1.6295 × 10^−2^	3.0578 × 10^−2^	−3.7656 × 10^−2^	−3.0030 × 10^−3^	1.41 × 10^−3^
−δ_2,PC_ (Å)	1.1080 × 10^−3^	3.9126 × 10^−4^	−3.4485 × 10^−4^	1.8136 × 10^−4^	–	4.81 × 10^−6^

## Footnotes

1 In order to generate the coordinates of Donnay & Le Page (1978 ▸), it would be necessary to shift the unit cell origin by  from the origin in *International Tables of X-ray Crystallography* (Hahn, 1995 ▸). The latter was adopted by Antao (2016 ▸).

2 The α_1_ and α_2_ structures are Dauphiné twins related by a 180° rotation about the threefold axis (Antao, 2016 ▸).

3 GeO_2_ was set in the *r*(+) setting according to Donnay and Le Page (1978 ▸). In order to generate the coordinates of Donnay and Le Page, it would be necessary to shift the unit cell origin by  from the origin in *International Tables of X-ray Crystallography* (Hahn, 1995 ▸). The latter was used by Haines *et al.* (2002 ▸).

4 Use of the concept of structural degrees of freedom has been made freely in earlier work (Thomas, 2017 ▸; Reifenberg & Thomas, 2018 ▸).

5 This is discussed in §S3 of the supporting information.

6 Values of *x*_0_ in equations (17) and (18) are in accordance with the notation in *International Tables for Crystallography* (Hahn, 1995 ▸) and equal to one half of the values of O*_x_* quoted by Antao (2016 ▸).

7 In a regular quartz, all four Si—O distances are equal. In α-quartz with distorted tetrahedra (Antao, 2016 ▸), there are two sets of two equal distances.

8 The observed decrease in Si—O distances with temperature in the experimental structures may an artefact arising from correlated thermal librations. Antao (2016 ▸) advocates a possible correction due to Downs *et al.* (1992 ▸).

## Appendix A

### A1. Curve-fitting coefficients for α-quartz

#### A1.1. Tilt angles ϕ_v_, ϕ_h_ and ϕ_m_

Polynomials of the form

were employed with *n* = 3 and δ calculated from equation (1). Fitting coefficients *a*_*i*_ are listed in Table 1 ▸.

#### A1.2. [Si+PC] parameters *L*, Δ, *a*_PC_, *b*_PC_ and *c*_PC_

Polynomials of the form of equation (A1.2) were employed with *n* = 3.

The reduced parameter applies here, as defined in equation (22). Fitting coefficients *a*_*i*_ are listed in Table 2 ▸.

### A2. Curve-fitting coefficients for β-quartz

Polynomials of the form of equation (*A*1.2) were employed with *n* = 4 for parameters *a*_PC_ and *b*_PC_ and *n* = 3 for parameter −δ_2,PC_. The reduced parameter applies here, as defined in equation (*A*2.1), whereby the temperatures of 860 K and 1235 K correspond to the minimum and maximum temperatures of the structures reported by Antao (2016 ▸) for the β-phase. Fitting coefficients *a*_*i*_ are listed in Table 7 ▸.

## References

[bb1] Alderson, A. & Evans, K. E. (2009). *J. Phys. Condens. Matter*, **21**, 025401. 10.1088/0953-8984/21/2/025401 21813974

[bb2] Antao, S. M. (2016). *Acta Cryst.* B**72**, 249–262. 10.1107/S205252061600233X 27048727

[bb3] Bärnighausen, H. (1980). *MATCH*, **9**, 139–175.

[bb4] Baumgartner, O., Behmer, M. & Preisinger, A. (1989). *Z. Kristallogr.* **187**, 125–131.

[bb5] Baumgartner, O., Preisinger, A., Krempl, P. W. & Mang, H. (1984). *Z. Kristallogr.* **168**, 83–91.

[bb6] Campbell, B. J., Stokes, H. T., Tanner, D. E. & Hatch, D. M. (2006). *J. Appl. Cryst.* **39**, 607–614.

[bb7] Carpenter, M. A., Salje, E. K. H., Graeme-Barber, A., Wruck, B., Dove, M. T. & Knight, K. S. (1998). *Am. Mineral.* **83**, 2–22.

[bb8] Dolino, G. (1990). *Phase Transit.* **21**, 59–72.

[bb9] Donnay, J. D. H. & Le Page, Y. (1978). *Acta Cryst.* A**34**, 584–594.

[bb10] Downs, R. T., Gibbs, C. V., Bartelmehs, K. L. & Boisen, M. B. Jr (1992). *Am. Mineral.* **77**, 751–757.

[bb11] Giddy, A. P., Dove, M. T., Pawley, G. S. & Heine, V. (1993). *Acta Cryst.* A**49**, 697–703.

[bb12] Glinnemann, J., King, H. E., Schulz, H., Hahn, T., Placa, S. J., La, & Dacol, F. (1992). *Z. Kristallogr.* **198**, 177–212.

[bb13] Grimm, H. & Dorner, B. (1975). *J. Phys. Chem. Solids*, **36**, 407–413.

[bb14] Hahn, T. (1995). Editor. *International Tables for Crystallography*, Vol. A, *Space-Group Symmetry.* Dordrecht: Kluwer.

[bb15] Haines, J., Cambon, O., Astier, R., Fertey, P. & Chateau, C. (2004). *Z. Kristallogr.* **219**, 32–37.

[bb16] Haines, J., Cambon, O. & Hull, S. (2003). *Z. Kristallogr.* **218**, 193–200.

[bb17] Haines, J., Cambon, O., Philippot, E., Chapon, L. & Hull, S. (2002). *J. Solid State Chem.* **166**, 434–441.

[bb18] Howard, C. J. & Stokes, H. T. (1998). *Acta Cryst.* B**54**, 782–789.

[bb19] Kihara, K. (1990). *Eur. J. Mineral.* **2**, 63–78.

[bb20] Kim-Zajonz, J., Werner, S. & Schulz, H. (1999). *Z. Kristallogr.* **214**, 324–330.

[bb21] Krempl, P. W. (2005). *J. Phys. IV Fr.* **126**, 95–100.

[bb22] Liu, L. & Bassett, W. A. (1986). *Elements, Oxides, Silicates. High-Pressure Phases with Implications for the Earth’s Interior*, p. 112. New York: Oxford University Press.

[bb23] Megaw, H. D. (1973*a*). *Crystal structures – a working approach*, p. 268. London: Saunders.

[bb24] Megaw, H. D. (1973*b*). *Crystal structures – a working approach*, pp. 453–456. London: Saunders.

[bb25] Nakae, H., Kihara, K., Okuno, M. & Hirano, S. (1995). *Z. Kristallogr.* **210**, 746–753.

[bb26] O’Keeffe, M. & Hyde, B. G. (1976). *Acta Cryst.* B**32**, 2923–2936.

[bb27] Reifenberg, M. & Thomas, N. W. (2018). *Acta Cryst.* B**74**, 165–181. 10.1107/S2052520618001312 29616992

[bb28] Smith, G. S. (1963). *Acta Cryst.* **16**, 542–545.

[bb29] Sowa, H. (1991). *Z. Kristallogr.* **194**, 291–304.

[bb30] Sowa, H. (1994). *Z. Kristallogr.* **209**, 954–960.

[bb31] Stokes, H. T. & Hatch, D. M. (1988). *Isotropy Subgroups of the 230 Crystallographic Space Groups.* Singapore: World Scientific.

[bb32] Tamazyan, R. & van Smaalen, S. (2007). *Acta Cryst.* B**63**, 190–200. 10.1107/S010876810605244X 17374928

[bb33] Taylor, D. (1984). *Mineral. Mag.* **48**, 65–79.

[bb34] Thomas, N. W. (1998). *Acta Cryst.* B**54**, 585–599.

[bb35] Thomas, N. W. (2017). *Acta Cryst.* B**73**, 74–86.

