# Supplementary material for: Modelling the structural variation of quartz and germanium dioxide with temperature by means of transformed crystallographic data
Source: Acta Crystallogr B Struct Sci Cryst Eng Mater. 2021 May 20;77(Pt 3):427–40. doi: 10.1107/S2052520621002717 (PMC8182799; doi:10.1107/S2052520621002717)
Supplement: Supplementary file 1 [file b-77-00427-sup1.pdf]

STRUCTURAL SCIENCE
CRYSTAL ENGINEERING
MATERIALS

Volume 77 (2021)

Supporting information for article:

Modelling the structural variation of quartz and germanium dioxide with temperature by means of transformed crystallographic data

Maximilian Fricke and Noel W. Thomas

Supporting information for the article entitled *Modelling the structural variation of quartz and germanium dioxide with temperature by means of transformed crystallographic data*

Maximilian Fricke & Noel W. Thomas
thomas@hs-koblenz.de

S1. Relationships between Si-network parameters L, Δ and crystallographic parameters *a* and x_{Si} in α-quartz and GeO_2

Reference may be made to Fig. 4 of the article. Lattice parameter *a* is equal to *p* + *q*. All the constitutive triangles making up the voids between Si-Si-Si equilateral triangles are congruent with side-lengths *L*, *p*, *q* and opposite angles of 60°, 60°-Δ and 60°+Δ, respectively.

Application of the sine rule leads to equation (S1.1).

$$\frac{L}{\sin 60°} = \frac{p}{\sin(60°-\Delta)} = \frac{q}{\sin(60°+\Delta)} \qquad \text{(S1.1)}$$

It follows that $p = \frac{L\sin(60°-\Delta)}{\sin 60°}$ and that $q = \frac{L\sin(60°+\Delta)}{\sin 60°}$. Further, lattice parameter *a* is equal to $\frac{L\sin(60°-\Delta) + L\sin(60°+\Delta)}{\sin 60°}$.

Use of a standard trigonometrical identity leads to the result $a = 2L\cos\Delta$. (S1.2)

The transformations of interest concern the values of network parameters *L* and Δ as a function of *a* and x_{Si} or x_{Ge}, simply denoted as *x* in the following treatment. *L* is the separation between two neighbouring Si or Ge ions. It may be derived from a difference vector between their coordinates in fractional coordinates that is subsequently transformed to Cartesian coordinates. Two neighbouring Si ions amongst the 3*a* positions in S.G. 154 are $\left[x, 0, \frac{2}{3}\right]$ and $[1-x, 1-x, 0]$. Collapsing these on to the *xy* plane, the difference vector $[1-2x, 1-x, 0]$ is obtained. Transformation into Cartesian coordinates by means of the orthogonalization matrix $\boldsymbol{O} = \begin{pmatrix} \frac{\sqrt{3}}{2}a & 0 & 0 \\ -\frac{a}{2} & a & 0 \\ 0 & 0 & c \end{pmatrix}$ leads to a Cartesian difference vector of $\begin{pmatrix} \frac{\sqrt{3}}{2}a(1-2x) \\ \frac{a}{2} \\ 0 \end{pmatrix}$.

Its modulus gives the value of *L* in terms of *a* and *x*, *i.e.*

$$L = a\sqrt{1-3x+3x^2} \qquad \text{(S1.3)}$$

It follows from equations (S1.2) and (S1.3) that $\Delta = \arccos\left(\frac{1}{2\sqrt{1-3x+3x^2}}\right)$. (S1.4)

<u>Calculation of results (S1.3) and (S1.4) with test data</u>

Antao (2016) gives the following data for α-quartz at 298 K:

a = 4.91339 Å; c = 5.40498 Å; silicon coordinates: x = 0.4719.

These yield the following results: L = 2.4683 Å; Δ = 5.56°.

S2. Analytical expressions for the pseudocubic parameters of O_4 tetrahedra and tetrahedral tilt angle in α-quartz and GeO_2

S2.1. Pseudocubic parameters

The formation of a pseudocube from its generating tetrahedron in α-quartz is shown in Fig. S1.

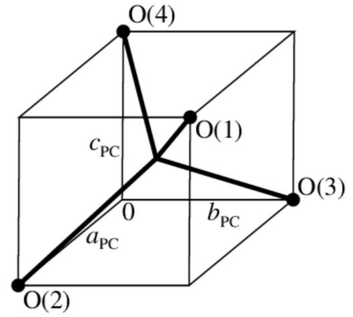

Figure S1 Ion O(1) is inverted at the centre-of-coordinates of the tetrahedron to define origin 0. Axis a_{PC} joins the origin to ion O(2), axis b_{PC} axis joins the origin to ion O(3) and axis c_{PC} the origin to ion O(4). Axes a_{PC}, b_{PC} and c_{PC} are chosen so that $a_{PC} \parallel x$ and c_{PC} lies closest to the z-axis. A right-handed set of vectors a_{PC}, b_{PC} and c_{PC} is formed in S.G. 154 and a left-handed set in the enantiomorphic S.G. 152.

Oxygen ions occupy positions 6c in space group $P3_221$ (S.G. 154) (Hahn, 1995):

$$(1)\ x,y,z;\ (2)\ \bar{y},x-y,z+\frac{2}{3};\ (3)\ \bar{x}+y,\bar{x},z+\frac{1}{3};\ (4)\ y,x,\bar{z};\ (5)\ x-y,\bar{y},\bar{z}+\frac{1}{3};\ (6)\ \bar{x},\bar{x}+y,\bar{z}+\frac{2}{3}$$

In accordance with Fig. S1, one of the three symmetrically equivalent O_4-tetrahedra in the unit cell, in this case the tetrahedron coordinating the silicon ion at $x_{Si},0,\frac{2}{3}$, may be formed as follows:

O(1): position (1); O(2): position (5) + lattice translation $[0,0,1]$; O(3): position (2) + lattice translation $[1,0,\bar{1}]$; O(4): position (6) + lattice translation $[1,0,1]$. (S2.1)

Similarly, oxygen ions occupy positions 6c in space group $P3_121$ (S.G. 152) (Hahn, 1995):

$$(1)\ x,y,z;\ (2)\ \bar{y},x-y,z+\frac{1}{3};\ (3)\ \bar{x}+y,\bar{x},z+\frac{2}{3};\ (4)\ y,x,\bar{z};\ (5)\ x-y,\bar{y},\bar{z}+\frac{2}{3};\ (6)\ \bar{x},\bar{x}+y,\bar{z}+\frac{1}{3}$$

One of the three symmetrically equivalent O_4-tetrahedra in the unit cell, in this case the tetrahedron coordinating the germanium ion at $x_{Ge},0,\frac{1}{3}$, may be formed as follows:

O(1): position (1); O(2): position (5); O(3): position (2) + lattice translation $[1,0,0]$; O(4): position (6) + lattice translation $[1,0,0]$. (S2.2)

The subsequent development proceeds in parallel for the enantiomorphic space groups group $P3_221$ and $P3_121$. The centre-of-inversion for the tetrahedron is given by the sum of the four sets of vertex coordinates divided by four, *i.e.*

$P3_221$	$P3_121$	
$\overline{x} = \frac{x+(x-y)+(1-y)+(1-x)}{4} = \frac{2+x-2y}{4}$; $\overline{y} = \frac{y-y+(x-y)+(-x+y)}{4} = 0$; $\overline{z} = \frac{z+\left(-z+\frac{4}{3}\right)+\left(z-\frac{1}{3}\right)+\left(-z+\frac{5}{3}\right)}{4} = \frac{2}{3}$	$\overline{x} = \frac{x+(x-y)+(1-y)+(1-x)}{4} = \frac{2+x-2y}{4}$; $\overline{y} = \frac{y-y+(x-y)+(-x+y)}{4} = 0$; $\overline{z} = \frac{z+\left(-z+\frac{2}{3}\right)+\left(z+\frac{1}{3}\right)+\left(-z+\frac{1}{3}\right)}{4} = \frac{1}{3}$	(S2.3)

The vector joining the centre-of-coordinates with oxygen ion O(1), which is arbitrarily assigned the symbol **v,** is given as follows in fractional coordinates:

$P3_221$	$P3_121$	
$\mathbf{v} = \begin{pmatrix} x \\ y \\ z \end{pmatrix} - \begin{pmatrix} \frac{2+x-2y}{4} \\ 0 \\ \frac{2}{3} \end{pmatrix} = \begin{pmatrix} \frac{3x+2y-2}{4} \\ y \\ z-\frac{2}{3} \end{pmatrix}$	$\mathbf{v} = \begin{pmatrix} x \\ y \\ z \end{pmatrix} - \begin{pmatrix} \frac{2+x-2y}{4} \\ 0 \\ \frac{1}{3} \end{pmatrix} = \begin{pmatrix} \frac{3x+2y-2}{4} \\ y \\ z-\frac{1}{3} \end{pmatrix}$	(S2.4)

The fractional coordinates of origin of the pseudocube, i.e. the inversion of oxygen ion O(1) at the centre-of-coordinates, is given by the following.

$P3_221$	$P3_121$	
$\mathbf{v_0} = \begin{pmatrix} \frac{2+x-2y}{4} \\ 0 \\ \frac{2}{3} \end{pmatrix} - \begin{pmatrix} \frac{3x+2y-2}{4} \\ y \\ z-\frac{2}{3} \end{pmatrix} = \begin{pmatrix} \frac{-x-2y+2}{2} \\ -y \\ \frac{4}{3}-z \end{pmatrix}$	$\mathbf{v_0} = \begin{pmatrix} \frac{2+x-2y}{4} \\ 0 \\ \frac{1}{3} \end{pmatrix} - \begin{pmatrix} \frac{3x+2y-2}{4} \\ y \\ z-\frac{1}{3} \end{pmatrix} = \begin{pmatrix} \frac{-x-2y+2}{2} \\ -y \\ \frac{2}{3}-z \end{pmatrix}$	(S2.5)

The three vectors corresponding to axes a_{PC}, b_{PC} and c_{PC} are as follows in fractional coordinates.

$P3_221$	$P3_121$
$\boldsymbol{a}_{PC} = \begin{pmatrix} x-y \\ -y \\ \frac{4}{3}-z \end{pmatrix} - \begin{pmatrix} \frac{-x-2y+2}{2} \\ -y \\ \frac{4}{3}-z \end{pmatrix} = \begin{pmatrix} \frac{3x}{2}-1 \\ 0 \\ 0 \end{pmatrix}$	$\boldsymbol{a}_{PC} = \begin{pmatrix} x-y \\ -y \\ \frac{2}{3}-z \end{pmatrix} - \begin{pmatrix} \frac{-x-2y+2}{2} \\ -y \\ \frac{2}{3}-z \end{pmatrix} = \begin{pmatrix} \frac{3x}{2}-1 \\ 0 \\ 0 \end{pmatrix}$
$\boldsymbol{b}_{PC} = \begin{pmatrix} 1-y \\ \mathrm{x}-y \\ -\frac{1}{3}+z \end{pmatrix} - \begin{pmatrix} \frac{-x-2y+2}{2} \\ -y \\ \frac{4}{3}-z \end{pmatrix} = \begin{pmatrix} \frac{x}{2} \\ x \\ 2z-\frac{5}{3} \end{pmatrix}$	$\boldsymbol{b}_{PC} = \begin{pmatrix} 1-y \\ \mathrm{x}-y \\ \frac{1}{3}+z \end{pmatrix} - \begin{pmatrix} \frac{-x-2y+2}{2} \\ -y \\ \frac{2}{3}-z \end{pmatrix} = \begin{pmatrix} \frac{x}{2} \\ x \\ 2z-\frac{1}{3} \end{pmatrix}$

$\boldsymbol{c}_{\mathrm{PC}} = \begin{pmatrix} 1-x \\ -x+y \\ \frac{5}{3}-z \end{pmatrix} - \begin{pmatrix} \frac{-x-2y+2}{2} \\ -y \\ \frac{4}{3}-z \end{pmatrix} = \begin{pmatrix} -\frac{x}{2}+y \\ -x+2y \\ \frac{1}{3} \end{pmatrix}$	$\boldsymbol{c}_{\mathrm{PC}} = \begin{pmatrix} 1-x \\ -x+y \\ \frac{1}{3}-z \end{pmatrix} - \begin{pmatrix} \frac{-x-2y+2}{2} \\ -y \\ \frac{2}{3}-z \end{pmatrix} = \begin{pmatrix} -\frac{x}{2}+y \\ -x+2y \\ -\frac{1}{3} \end{pmatrix}$

(S2.6)

A transformation of these vectors into Cartesian coordinates is carried out by means of the following orthogonalization matrix:

$$\boldsymbol{O} = \begin{pmatrix} \frac{\sqrt{3}}{2}a & 0 & 0 \\ -\frac{a}{2} & a & 0 \\ 0 & 0 & c \end{pmatrix} \quad \text{(S2.7)}$$

Pre-multiplication of the three vectors in equations (S2.6) with this matrix leads to the following vectors in Cartesian coordinates:

$P3_221$	$P3_121$
$\boldsymbol{a}_{\mathrm{PC}}^{C} = \begin{pmatrix} \frac{\sqrt{3}}{2}a\left(\frac{3x}{2}-1\right) \\ -\frac{a}{2}\left(\frac{3x}{2}-1\right) \\ 0 \end{pmatrix}$	$\boldsymbol{a}_{\mathrm{PC}}^{C} = \begin{pmatrix} \frac{\sqrt{3}}{2}a\left(\frac{3x}{2}-1\right) \\ -\frac{a}{2}\left(\frac{3x}{2}-1\right) \\ 0 \end{pmatrix}$
$\boldsymbol{b}_{\mathrm{PC}}^{C} = \begin{pmatrix} \frac{\sqrt{3}}{4}ax \\ \frac{3ax}{4} \\ c\left(2z-\frac{5}{3}\right) \end{pmatrix}$	$\boldsymbol{b}_{\mathrm{PC}}^{C} = \begin{pmatrix} \frac{\sqrt{3}}{4}ax \\ \frac{3ax}{4} \\ c\left(2z-\frac{1}{3}\right) \end{pmatrix}$
$\boldsymbol{c}_{\mathrm{PC}}^{C} = \begin{pmatrix} \frac{\sqrt{3}}{4}a(-x+2y) \\ \frac{3a}{4}(-x+2y) \\ \frac{c}{3} \end{pmatrix}$	$\boldsymbol{c}_{\mathrm{PC}}^{C} = \begin{pmatrix} \frac{\sqrt{3}}{4}a(-x+2y) \\ \frac{3a}{4}(-x+2y) \\ -\frac{c}{3} \end{pmatrix}$

(S2.8)

The lengths of axes a_{PC}. b_{PC} and c_{PC} are given by the moduli of these three vectors, *i.e.*

$P3_221$	$P3_121$
$a_{\mathrm{PC}} = \left\lvert a\left(\frac{3x}{2}-1\right)\right\rvert$	$a_{\mathrm{PC}} = \left\lvert a\left(\frac{3x}{2}-1\right)\right\rvert$
$b_{\mathrm{PC}} = \sqrt{\frac{3}{4}a^2x^2 + c^2\left(2z-\frac{5}{3}\right)^2}$	$b_{\mathrm{PC}} = \sqrt{\frac{3}{4}a^2x^2 + c^2\left(2z-\frac{1}{3}\right)^2}$

(S2.9)

$c_{\mathrm{PC}} = \sqrt{\frac{3a^2}{4}(-x+2y)^2 + \frac{c^2}{9}}$	$c_{\mathrm{PC}} = \sqrt{\frac{3a^2}{4}(-x+2y)^2 + \frac{c^2}{9}}$

The angles between the three axes are determined by the three scalar products $\boldsymbol{b}_{\mathrm{PC}}^{C} \cdot \boldsymbol{c}_{\mathrm{PC}}^{C}$, $\boldsymbol{c}_{\mathrm{PC}}^{C} \cdot \boldsymbol{a}_{\mathrm{PC}}^{C}$ and $\boldsymbol{a}_{\mathrm{PC}}^{C} \cdot \boldsymbol{b}_{\mathrm{PC}}^{C}$, whereby only the first of these is non-zero:

*P*3$_2$21	*P*3$_1$21
$\boldsymbol{b}_{\mathrm{PC}}^{C} \cdot \boldsymbol{c}_{\mathrm{PC}}^{C} = \frac{1}{3}c^2\left(2z - \frac{5}{3}\right) - \frac{3}{4}a^2(x^2 - 2xy)$	$\boldsymbol{b}_{\mathrm{PC}}^{C} \cdot \boldsymbol{c}_{\mathrm{PC}}^{C} = -\frac{1}{3}c^2\left(2z - \frac{1}{3}\right) - \frac{3}{4}a^2(x^2 - 2xy)$
$\alpha_{\mathrm{PC}} = \arccos\left(\frac{\frac{1}{3}c^2\left(2z - \frac{5}{3}\right) - \frac{3}{4}a^2(x^2 - 2xy)}{b_{\mathrm{PC}}c_{\mathrm{PC}}}\right)$	$\alpha_{\mathrm{PC}} = \arccos\left(\frac{-\frac{1}{3}c^2\left(2z - \frac{1}{3}\right) - \frac{3}{4}a^2(x^2 - 2xy)}{b_{\mathrm{PC}}c_{\mathrm{PC}}}\right)$
$\beta_{\mathrm{PC}} = \gamma_{\mathrm{PC}} = 90°$	$\beta_{\mathrm{PC}} = \gamma_{\mathrm{PC}} = 90°$ (S2.10)

Calculation of results (S2.8), (S2.9) and (S2.10) with test data

*P*3$_2$21	*P*3$_1$21
α-quartz at 298 K (Antao, 2016)<br>*a* = 4.91339 Å; *c* = 5.40498 Å<br>oxygen coordinates: *x* = 0.4125; *y* = 0.2648; *z* = 0.7874	α-GeO_2 at 294 K (Haines *et al.*, 2002)<br>*a* = 4.98503 Å; *c* = 5.64711 Å<br>oxygen coordinates: *x* = 0.3974; *y* = 0.3022; *z* = 0.2425
$\boldsymbol{a}_{\mathrm{PC}}^{C} = \begin{pmatrix}-1.6223\\0.9366\\0\end{pmatrix}$ Å; $\boldsymbol{b}_{\mathrm{PC}}^{C} = \begin{pmatrix}0.8776\\1.5201\\-0.4965\end{pmatrix}$ Å<br>$\boldsymbol{c}_{\mathrm{PC}}^{C} = \begin{pmatrix}0.2491\\0.4315\\1.8017\end{pmatrix}$ Å	$\boldsymbol{a}_{\mathrm{PC}}^{C} = \begin{pmatrix}-1.7437\\1.0067\\0\end{pmatrix}$ Å; $\boldsymbol{b}_{\mathrm{PC}}^{C} = \begin{pmatrix}0.8578\\1.4858\\0.8565\end{pmatrix}$ Å<br>$\boldsymbol{c}_{\mathrm{PC}}^{C} = \begin{pmatrix}0.4468\\0.7739\\-1.8824\end{pmatrix}$ Å
$a_{\mathrm{PC}} = 1.8732$ Å; $b_{\mathrm{PC}} = 1.8241$ Å;<br>$c_{\mathrm{PC}} = 1.8693$ Å; $\alpha_{\mathrm{PC}} = 90.34°$	$a_{\mathrm{PC}} = 2.0135$ Å; $b_{\mathrm{PC}} = 1.9175$ Å;<br>$c_{\mathrm{PC}} = 2.0837$ Å; $\alpha_{\mathrm{PC}} = 91.13°$

S2.2. Calculation of tetrahedral tilt angle

Fig. 3 in the article shows tilt angles ϕ_h and ϕ_v of the oxygen pseudocubes in both enantiomeric space groups. The left-hand diagram correlates with Fig. 2 in the article. The subscript h or v indicates whether the reference direction is horizontal, *i.e.* ⊥ *z*-axis in the *xy*-plane, or vertical, *i.e.* || *z*. Since angle α_{PC} deviates from 90°, ϕ_h is only approximately equal to ϕ_v. Therefore a mean tilt angle $\phi = \frac{\phi_h + \phi_v}{2}$ may be taken.

The magnitude of ϕ_h is given by the scalar product of vector $\boldsymbol{b}_{\mathrm{PC}}^{C}$ (equations (S2.8)) and its projection in the *xy* plane. It is seen in the following that the same result applies to both space groups.

	$P3_221$	$P3_121$
Vector $\boldsymbol{b}_{\mathrm{PC}}^{C}$<br>Magnitude: b_{PC}	$\boldsymbol{b}_{\mathrm{PC}}^{C} = \begin{pmatrix} \frac{\sqrt{3}}{4}ax \\ \frac{3ax}{4} \\ c\left(2z - \frac{5}{3}\right) \end{pmatrix}$	$\boldsymbol{b}_{\mathrm{PC}}^{C} = \begin{pmatrix} \frac{\sqrt{3}}{4}ax \\ \frac{3ax}{4} \\ c\left(2z - \frac{1}{3}\right) \end{pmatrix}$
Projection of vector $\boldsymbol{b}_{\mathrm{PC}}^{C}$ in *xy* plane<br>Magnitude: $\frac{\sqrt{3}}{2}ax$	$\begin{pmatrix} \frac{\sqrt{3}}{4}ax \\ \frac{3ax}{4} \\ 0 \end{pmatrix}$	$\begin{pmatrix} \frac{\sqrt{3}}{4}ax \\ \frac{3ax}{4} \\ 0 \end{pmatrix}$
Scalar product:	$\frac{3}{4}a^2x^2$	$\frac{3}{4}a^2x^2$
ϕ_h	$\arccos\left(\frac{\sqrt{3}ax}{2b_{\mathrm{PC}}}\right)$	$\arccos\left(\frac{\sqrt{3}ax}{2b_{\mathrm{PC}}}\right)$

(S2.11)

The magnitude of ϕ_v is given by the scalar product of vector $\boldsymbol{c}_{\mathrm{PC}}^{C}$ (equations (S2.8)) and its projection along the positive or negative *z*-axis, according to Fig. 3 in the article. It is seen in the following that the same result applies to both space groups.

	$P3_221$	$P3_121$
Vector $\boldsymbol{c}_{\mathrm{PC}}^{C}$<br>Magnitude: c_{PC}	$\boldsymbol{c}_{\mathrm{PC}}^{C} = \begin{pmatrix} \frac{\sqrt{3}}{4}a(-x+2y) \\ \frac{3a}{4}(-x+2y) \\ \frac{c}{3} \end{pmatrix}$	$\boldsymbol{c}_{\mathrm{PC}}^{C} = \begin{pmatrix} \frac{\sqrt{3}}{4}a(-x+2y) \\ \frac{3a}{4}(-x+2y) \\ -\frac{c}{3} \end{pmatrix}$
Projection of vector $\boldsymbol{c}_{\mathrm{PC}}^{C}$ $\parallel z$	$\begin{pmatrix} 0 \\ 0 \\ \frac{c}{3} \end{pmatrix}$	$\begin{pmatrix} 0 \\ 0 \\ -\frac{c}{3} \end{pmatrix}$
Scalar product:	$\frac{c^2}{9}$	$\frac{c^2}{9}$
ϕ_v	$\phi_{\mathrm{v}} = \arccos\left(\frac{c}{3c_{\mathrm{PC}}}\right)$	$\phi_{\mathrm{v}} = \arccos\left(\frac{c}{3c_{\mathrm{PC}}}\right)$

(S2.12)

S3. Analytical expressions for the pseudocubic parameters of O_4 tetrahedra in β-quartz

S3.1. Parameters a_{PC}, b_{PC} and c_{PC}

The following derivations follow the same logic as for α-quartz in §S2.1.

Oxygen ions occupy positions 6*j* in S.G. 180 (Hahn, 1995):

$$(1)\ x, 2x, \tfrac{1}{2};\ (2)\ 2\bar{x}, \bar{x}, \tfrac{1}{6};\ (3)\ x, \bar{x}, \tfrac{5}{6};\ (4)\ \bar{x}, 2\bar{x}, \tfrac{1}{2};\ (5)\ 2x, x, \tfrac{1}{6};\ (6)\ \bar{x}, x, \tfrac{5}{6}\,.$$

In accordance with Fig. S1, one of the three symmetrically equivalent O_4-tetrahedra in the unit cell, in this case the tetrahedron coordinating the silicon ion at $\frac{1}{2}$,0,0, may be formed as follows:

O(1): position (5); O(2): position (6) + lattice translation $[1,0,\bar{1}]$; O(3): position (3) + lattice translation $[0,0,\bar{1}]$; O(4): position (2) + lattice translation $[1,0,0]$. (S3.1)

The centre-of-coordinates of the tetrahedron is given by the sum of the four sets of vertex coordinates divided by four, *i.e.*

$$\bar{x} = \frac{2x+(1-x)+x+(1-2x)}{4} = \frac{1}{2};\ \ \bar{y} = \frac{x+x-x-x}{4} = 0\ ;\ \bar{z} = \frac{\frac{1}{6}+\left(\frac{5}{6}-1\right)+\left(\frac{5}{6}-1\right)+\frac{1}{6}}{4} = 0\ . \quad \text{(S3.2)}$$

The vector joining the centre-of-coordinates with oxygen ion O(1), which is arbitrarily assigned the symbol **v,** is given as follows in fractional coordinates:

$$\mathbf{v} = \begin{pmatrix} 2x \\ x \\ \frac{1}{6} \end{pmatrix} - \begin{pmatrix} \frac{1}{2} \\ 0 \\ 0 \end{pmatrix} = \begin{pmatrix} \frac{4x-1}{2} \\ x \\ \frac{1}{6} \end{pmatrix} \qquad \text{(S3.3)}$$

The fractional coordinates of origin of the pseudocube, i.e. the inversion of oxygen ion O(1) at the centre-of-coordinates, is given by

$$\mathbf{v_0} = \begin{pmatrix} \frac{1}{2} \\ 0 \\ 0 \end{pmatrix} - \begin{pmatrix} \frac{4x-1}{2} \\ x \\ \frac{1}{6} \end{pmatrix} = \begin{pmatrix} 1-2x \\ -x \\ -\frac{1}{6} \end{pmatrix} \qquad \text{(S3.4)}$$

Here, $\mathbf{v}_0$ represents the coordinates of the origin of the pseudocubic cell, 0, in the fractional coordinate system of the four oxygen ions. The three vectors corresponding to axes a_{PC}, b_{PC} and c_{PC} are as follows in fractional coordinates.

$$\boldsymbol{a}_{\mathrm{PC}} = \begin{pmatrix} 1-x \\ \mathrm{x} \\ -\frac{1}{6} \end{pmatrix} - \begin{pmatrix} 1-2x \\ -x \\ -\frac{1}{6} \end{pmatrix} = \begin{pmatrix} x \\ 2x \\ 0 \end{pmatrix}$$

$$\boldsymbol{b}_{\mathrm{PC}} = \begin{pmatrix} x \\ -x \\ -\frac{1}{6} \end{pmatrix} - \begin{pmatrix} 1-2x \\ -x \\ -\frac{1}{6} \end{pmatrix} = \begin{pmatrix} 3x-1 \\ 0 \\ 0 \end{pmatrix} \qquad \text{(S3.5)}$$

$$\boldsymbol{c}_{\mathrm{PC}} = \begin{pmatrix} 1-2x \\ -x \\ \frac{1}{6} \end{pmatrix} - \begin{pmatrix} 1-2x \\ -x \\ -\frac{1}{6} \end{pmatrix} = \begin{pmatrix} 0 \\ 0 \\ \frac{1}{3} \end{pmatrix}$$

Pre-multiplication of the three vectors in equations (S3.5) with orthogonalization matrix (S2.7) leads to the following vectors in Cartesian coordinates:

$$\boldsymbol{a}_{\mathrm{PC}}^{C} = \begin{pmatrix} \frac{\sqrt{3}}{2}ax \\ \frac{3}{2}ax \\ 0 \end{pmatrix} \qquad \boldsymbol{b}_{\mathrm{PC}}^{C} = \begin{pmatrix} \frac{\sqrt{3}}{2}a(3x-1) \\ -\frac{1}{2}a(3x-1) \\ 0 \end{pmatrix} \qquad \boldsymbol{c}_{\mathrm{PC}}^{C} = \begin{pmatrix} 0 \\ 0 \\ \frac{c}{3} \end{pmatrix} \qquad \text{(S3.6)}$$

The lengths of axes a_{PC}, b_{PC} and c_{PC} are given by the moduli of these three vectors, *i.e.*

$$a_{\mathrm{PC}} = \sqrt{3}ax \qquad b_{\mathrm{PC}} = |a(3x-1)| \qquad c_{\mathrm{PC}} = \frac{c}{3} \qquad \text{(S3.7)}$$

The angles between the three axes are determined by the three scalar products $\boldsymbol{b}_{\mathrm{PC}}^{C} \cdot \boldsymbol{c}_{\mathrm{PC}}^{C}$, $\boldsymbol{c}_{\mathrm{PC}}^{C} \cdot \boldsymbol{a}_{\mathrm{PC}}^{C}$ and $\boldsymbol{a}_{\mathrm{PC}}^{C} \cdot \boldsymbol{b}_{\mathrm{PC}}^{C}$, which are all equal to zero.

It follows that $\alpha_{\mathrm{PC}} = \beta_{\mathrm{PC}} = \gamma_{\mathrm{PC}} = 90°$. (S3.8)

<u>Calculation of results (S3.7) with test data</u>

Antao (2016) gives the following data for β-quartz at 875 K:

a = 4.9957 Å; *c* = 5.4563 Å; oxygen coordinates: 2*x* = 0.4187.

These yield the following values for the lengths of the pseudocube axes:

a_{PC} = 1.8115 Å; b_{PC} = 1.8582 Å; c_{PC} = 1.8188 Å.

S3.2. Special handling of deviations from the ideal of regular tetrahedra for β-quartz

The structure of β-quartz is determined by just three parameters, *a*, *c* and *x*, the latter referring to the oxygen ion. Furthermore, all pseudocubic angles are equal to 90° (equation (S3.8)). With respect to the oxygen tetrahedra or pseudocubes, one of the three degrees of freedom is taken up with expressing pseudocube volume, with the remaining two relating to pseudocubic form.

For a regular pseudocube, *i.e.* a perfect cube, $a_{\mathrm{PC}} = b_{\mathrm{PC}} = c_{\mathrm{PC}}$. If unit cell parameter *a* is regarded as a scaling parameter with no influence on pseudocubic form, equations (S3.7) take on the following reduced form.

$$\frac{a_{\mathrm{PC}}}{a} = \sqrt{3}x \qquad \frac{b_{\mathrm{PC}}}{a} = |(3x-1)| \qquad \frac{c_{\mathrm{PC}}}{a} = \frac{1}{3}\frac{c}{a} \qquad \text{(S3.9)}$$

The criterion $\frac{a_{\mathrm{PC}}}{a} = \frac{b_{\mathrm{PC}}}{a}$ demands that $\sqrt{3}x = |(3x-1)|$, which on squaring, leads to the quadratic equation $6x^2 - 6x + 1 = 0$. Its two roots are $x = \frac{1}{2} \pm \frac{\sqrt{3}}{6}$, of which the root with negative sign applies to

the Antao (2016) solutions, *i.e.* $x_\mathrm{C} = \frac{1}{2} - \frac{\sqrt{3}}{6} \approx 0.21132$. Here x_C stands for the unique value of x leading a_PC being equal to b_PC. In the case of a perfect cube, values of c_PC and a_PC will be also be equal, such that $\frac{c_\mathrm{PC}}{\sqrt{3}a} = \frac{a_\mathrm{PC}}{\sqrt{3}a} = x_\mathrm{C}$. Since the factor $\frac{c_\mathrm{PC}}{\sqrt{3}a}$ can be re-expressed as $\frac{\sqrt{3}}{9}\frac{c}{a}$, it follows that the two parameters $(x - x_\mathrm{C})$ and $\left(\frac{\sqrt{3}}{9}\frac{c}{a} - x_\mathrm{C}\right)$ provide independent indicators of deviations from perfect cubicity and, by implication, tetrahedral regularity. Both parameters are equal to zero for a regular tetrahedron. They are assigned the symbols $\delta_{1,\mathrm{PC}}$ and $\delta_{2,\mathrm{PC}}$ in the article.

S4. Calculated silicon network and oxygen pseudocube parameters for α-quartz, GeO_2 and β-quartz

The order of results in Table S1 follows Table 1 of Antao (2016). SiO_4-tetrahedral volumes, V_{tetra}, were calculated from oxygen pseudocubic parameters following equation (1) of Reifenberg and Thomas (2018).

Table S1 Calculated parameters for α-quartz. Parameters in columns 2 to 7 are calculated from equations (2),(3),(6),(7),(9) and (10) of the article. Columns 8, 9 and 10 contain unit cell volumes, SiO_4 tetrahedral volumes and the fractional space occupied by SiO_4-tetrahedra, respectively. Column 11 contains values of λ_{PC}, the length-based tetrahedral distortion parameter (Reifenberg and Thomas, 2018). Columns 12 to 13 contain pseudocubic tilt angles ϕ, as defined in equations (13) and (14) of the article.

T(K)	L(Å)	Δ(°)	a_{PC}(Å)	b_{PC}(Å)	c_{PC}(Å)	α_{PC}(°)	V_{UC}(Å^3)	V_{tetra}(Å^3)	$3V_{tetra}/V_{UC}$	λ_{PC}	ϕ_v(°)	ϕ_h(°)
298	2.4683(2)	5.56(4)	1.873(1)	1.824(1)	1.8693(5)	90.34(8)	113.005(3)	2.135(2)	0.0567(1)	0.0113(4)	15.46(6)	15.80(7)
298	2.4683(2)	5.56(4)	1.873(1)	1.824(1)	1.8695(5)	90.31(8)	113.002(3)	2.135(2)	0.0567(1)	0.0113(4)	15.48(6)	15.80(7)
334	2.4692(2)	5.48(4)	1.872(1)	1.825(1)	1.8679(5)	90.40(8)	113.141(3)	2.135(2)	0.0566(1)	0.0108(4)	15.25(6)	15.65(7)
337	2.4693(2)	5.48(4)	1.872(1)	1.824(1)	1.8677(5)	90.33(8)	113.152(3)	2.132(2)	0.0565(1)	0.0109(4)	15.22(6)	15.55(7)
324	2.4690(2)	5.52(4)	1.872(1)	1.825(1)	1.8684(5)	90.36(8)	113.099(3)	2.134(2)	0.0566(1)	0.0108(4)	15.32(6)	15.68(7)
322	2.4689(2)	5.52(4)	1.872(1)	1.825(1)	1.8682(5)	90.38(8)	113.089(3)	2.134(2)	0.0566(1)	0.0109(4)	15.31(6)	15.69(7)
323	2.4689(2)	5.52(4)	1.873(1)	1.824(1)	1.8686(5)	90.34(8)	113.092(3)	2.135(2)	0.0566(1)	0.0112(4)	15.35(6)	15.69(7)
323	2.4690(2)	5.54(4)	1.873(1)	1.824(1)	1.8681(5)	90.39(8)	113.090(3)	2.135(2)	0.0566(1)	0.0111(4)	15.30(6)	15.69(7)
329	2.4691(2)	5.52(4)	1.872(1)	1.824(1)	1.8681(5)	90.34(8)	113.113(3)	2.133(2)	0.0566(1)	0.0111(4)	15.29(6)	15.62(7)
345	2.4694(2)	5.46(4)	1.872(1)	1.824(1)	1.8673(5)	90.38(8)	113.175(3)	2.133(2)	0.0565(1)	0.0109(4)	15.17(6)	15.55(7)

362	2.4698(2)	5.42(4)	1.872(1)	1.824(1)	1.8666(5)	90.39(8)	113.244(3)	2.132(2)	0.0565(1)	0.0108(4)	15.06(6)	15.45(7)
378	2.4702(2)	5.38(4)	1.872(1)	1.824(1)	1.8659(5)	90.39(8)	113.309(3)	2.131(2)	0.0564(1)	0.0108(4)	14.96(6)	15.35(7)
394	2.4706(2)	5.34(4)	1.871(1)	1.824(1)	1.8653(5)	90.38(8)	113.375(3)	2.129(2)	0.0563(1)	0.0106(4)	14.86(6)	15.24(7)
410	2.4710(2)	5.30(4)	1.871(1)	1.824(1)	1.8646(5)	90.35(8)	113.440(3)	2.127(2)	0.0563(1)	0.0105(4)	14.75(6)	15.11(7)
426	2.4715(2)	5.26(4)	1.871(1)	1.824(1)	1.8640(5)	90.39(8)	113.511(3)	2.128(2)	0.0562(1)	0.0105(4)	14.65(6)	15.04(7)
441	2.4719(2)	5.23(4)	1.870(1)	1.824(1)	1.8631(5)	90.37(9)	113.576(3)	2.125(2)	0.0561(1)	0.0103(4)	14.53(6)	14.90(7)
457	2.4723(2)	5.17(4)	1.871(1)	1.824(1)	1.8626(5)	90.41(9)	113.651(3)	2.126(2)	0.0561(1)	0.0103(4)	14.43(6)	14.83(7)
473	2.4726(2)	5.09(4)	1.871(1)	1.824(1)	1.8615(5)	90.43(9)	113.726(3)	2.125(2)	0.0560(1)	0.0102(4)	14.26(6)	14.69(7)
489	2.4731(2)	5.05(4)	1.871(1)	1.823(1)	1.8605(5)	90.45(9)	113.801(3)	2.124(2)	0.0560(1)	0.0103(4)	14.11(6)	14.56(7)
504	2.4735(2)	4.99(4)	1.870(1)	1.823(1)	1.8601(5)	90.35(9)	113.871(3)	2.120(2)	0.0559(1)	0.0101(4)	14.04(6)	14.39(7)
520	2.4740(1)	4.95(4)	1.871(1)	1.823(1)	1.8596(5)	90.35(9)	113.948(3)	2.120(2)	0.0558(1)	0.0102(4)	13.94(6)	14.29(7)
535	2.4745(1)	4.91(4)	1.870(1)	1.822(1)	1.8591(5)	90.30(9)	114.028(3)	2.118(2)	0.0557(1)	0.0102(4)	13.85(6)	14.15(7)
551	2.4751(1)	4.85(4)	1.869(1)	1.823(1)	1.8583(5)	90.31(9)	114.120(3)	2.116(2)	0.0556(1)	0.0099(4)	13.70(6)	14.01(7)
566	2.4755(1)	4.77(4)	1.869(1)	1.823(1)	1.8575(4)	90.32(9)	114.205(3)	2.115(2)	0.0556(1)	0.0098(4)	13.56(6)	13.87(7)
582	2.4761(1)	4.71(4)	1.868(1)	1.823(1)	1.8570(4)	90.24(9)	114.298(3)	2.112(2)	0.0554(1)	0.0096(4)	13.46(6)	13.70(7)
597	2.4768(1)	4.67(4)	1.869(1)	1.822(1)	1.8568(6)	90.15(10)	114.394(3)	2.110(2)	0.0553(1)	0.0098(4)	13.38(8)	13.53(7)
612	2.4772(1)	4.58(4)	1.871(1)	1.821(1)	1.8562(6)	90.15(10)	114.488(3)	2.110(2)	0.0553(1)	0.0103(4)	13.25(8)	13.40(7)
628	2.4779(1)	4.52(4)	1.869(1)	1.821(1)	1.8552(6)	90.08(10)	114.590(3)	2.106(2)	0.0551(1)	0.0098(4)	13.08(8)	13.16(7)

644	2.4784(1)	4.42(4)	1.869(1)	1.821(1)	1.8547(6)	89.99(13)	114.692(3)	2.103(3)	0.0550(1)	0.0098(4)	12.96(8)	12.95(10)
660	2.4791(2)	4.34(6)	1.869(1)	1.820(1)	1.8535(6)	89.97(13)	114.810(3)	2.101(3)	0.0549(1)	0.0098(4)	12.74(8)	12.71(10)
676	2.4798(2)	4.26(6)	1.868(1)	1.820(1)	1.8528(6)	89.88(13)	114.920(3)	2.097(3)	0.0548(1)	0.0097(4)	12.59(8)	12.47(10)
691	2.4804(2)	4.14(6)	1.867(1)	1.820(1)	1.8522(6)	89.79(13)	115.042(3)	2.094(3)	0.0546(1)	0.0095(4)	12.44(8)	12.23(10)
706	2.4811(2)	4.08(6)	1.866(1)	1.820(1)	1.8517(6)	89.68(13)	115.151(3)	2.091(3)	0.0545(1)	0.0094(4)	12.31(8)	11.99(10)
722	2.4819(2)	3.94(6)	1.865(1)	1.820(1)	1.8503(6)	89.64(13)	115.294(4)	2.087(3)	0.0543(1)	0.0091(4)	12.04(8)	11.68(10)
737	2.4827(2)	3.82(6)	1.864(1)	1.820(1)	1.8495(6)	89.55(13)	115.428(4)	2.083(3)	0.0541(1)	0.0087(4)	11.85(8)	11.40(10)
752	2.4835(2)	3.71(6)	1.864(1)	1.820(1)	1.8483(7)	89.49(15)	115.566(4)	2.080(3)	0.0540(1)	0.0088(4)	11.60(11)	11.09(10)
768	2.4843(2)	3.49(6)	1.864(1)	1.820(1)	1.8475(7)	89.44(15)	115.740(4)	2.078(3)	0.0539(1)	0.0087(4)	11.38(11)	10.82(10)
784	2.4854(2)	3.37(6)	1.863(1)	1.819(1)	1.8463(7)	89.26(15)	115.899(4)	2.072(3)	0.0536(1)	0.0085(4)	11.11(11)	10.37(10)
798	2.4864(1)	3.15(6)	1.863(1)	1.818(1)	1.8444(7)	89.17(17)	116.093(4)	2.067(4)	0.0534(1)	0.0085(4)	10.69(11)	9.86(14)
813	2.4876(1)	2.92(6)	1.862(1)	1.818(1)	1.8430(6)	89.03(17)	116.305(4)	2.061(4)	0.0532(1)	0.0082(4)	10.32(11)	9.34(14)
829	2.4891(1)	2.42(8)	1.862(1)	1.816(1)	1.8405(6)	88.77(17)	116.603(4)	2.052(3)	0.0528(1)	0.0084(4)	9.69(11)	8.45(14)
844	2.4907(1)	1.92(8)	1.859(1)	1.816(1)	1.8375(7)	88.53(22)	116.894(4)	2.042(4)	0.0524(1)	0.0078(5)	8.92(14)	7.45(17)

Table S2 Calculated parameters for GeO_2. Parameters in columns 2 to 7 are calculated from equations (2),(3),(6),(8),(9) and (11) of the article. Columns 8, 9 and 10 contain unit cell volumes, GeO_4 tetrahedral volumes and the fractional space occupied by GeO_4-tetrahedra, respectively. Column 11 contains values of λ_{PC}, the length-based tetrahedral distortion parameter (Reifenberg and Thomas, 2018). Columns 12 to 13 contain pseudocubic tilt angles ϕ, as defined in equations (13) and (14) of the article.

T(K)	L(Å)	Δ(°)	a_{PC}(Å)	b_{PC}(Å)	c_{PC}(Å)	α_{PC}(°)	V_{UC}(Å^3)	V_{tetra}(Å^3)	$3V_{tetra}/V_{UC}$	λ_{PC}	ϕ_v(°)	ϕ_h(°)
294	2.5280(1)	9.61(2)	2.013(1)	1.918(1)	2.084(0)	91.13(4)	121.532(2)	2.708(2)	0.0668(0)	0.0291(2)	25.40(2)	26.53(3)
298	2.5280(1)	9.60(2)	2.016(1)	1.918(1)	2.084(1)	91.17(4)	121.503(1)	2.713(2)	0.0670(0)	0.0293(4)	25.46(3)	26.63(3)
425	2.5306(4)	9.31(6)	2.020(3)	1.916(2)	2.081(1)	91.11(9)	122.039(3)	2.710(4)	0.0666(1)	0.0299(8)	25.20(8)	26.30(6)
449	2.5323(1)	9.50(2)	2.002(1)	1.932(1)	2.081(1)	91.43(7)	122.111(2)	2.716(3)	0.0667(1)	0.0252(2)	25.13(5)	26.56(6)
571	2.5352(4)	9.25(6)	2.020(3)	1.918(2)	2.077(2)	91.18(13)	122.611(3)	2.710(5)	0.0663(1)	0.0289(8)	24.88(10)	26.06(9)
634	2.5370(3)	9.23(4)	2.003(2)	1.934(1)	2.077(1)	91.46(7)	122.887(3)	2.716(3)	0.0663(1)	0.0240(2)	24.77(5)	26.22(6)
756	2.5404(5)	9.00(8)	2.020(4)	1.920(2)	2.072(2)	91.19(14)	123.405(4)	2.705(5)	0.0658(1)	0.0280(10)	24.42(12)	25.62(10)
845	2.5435(3)	8.96(4)	2.005(2)	1.935(2)	2.072(1)	91.41(8)	123.818(3)	2.713(3)	0.0657(1)	0.0229(5)	24.34(5)	25.75(6)
890	2.5440(5)	8.76(8)	2.020(5)	1.918(3)	2.065(2)	91.17(18)	123.999(4)	2.696(7)	0.0652(2)	0.0277(14)	23.96(15)	25.13(13)
1043	2.5504(8)	8.43(12)	2.023(7)	1.922(4)	2.058(3)	91.25(23)	124.806(4)	2.696(8)	0.0648(2)	0.0263(18)	23.53(19)	24.77(16)
1059	2.5521(3)	8.69(4)	2.020(2)	1.928(2)	2.062(1)	91.26(9)	124.844(2)	2.705(3)	0.0650(1)	0.0251(6)	23.74(7)	25.00(6)
1175	2.5555(3)	8.43(4)	2.028(3)	1.923(2)	2.055(1)	91.36(9)	125.389(2)	2.702(4)	0.0646(1)	0.0263(8)	23.23(8)	24.59(7)
1215	2.5563(4)	8.43(6)	2.034(4)	1.915(2)	2.051(2)	91.30(13)	125.469(3)	2.693(5)	0.0644(1)	0.0284(10)	22.99(11)	24.29(10)
1275	2.5597(7)	8.12(12)	2.042(8)	1.914(5)	2.046(3)	91.50(27)	126.043(4)	2.701(10)	0.0643(2)	0.0288(22)	22.62(22)	24.12(20)

1298	2.5595(4)	8.22(6)	2.028(4)	1.922(2)	2.045(2)	91.60(13)	125.972(3)	2.694(5)	0.0642(1)	0.0254(10)	22.53(11)	24.13(10)
1344	2.5607(2)	8.10(4)	2.036(4)	1.914(2)	2.043(2)	91.36(13)	126.174(3)	2.684(5)	0.0638(1)	0.0279(10)	22.36(11)	23.73(10)

Table S3 Calculated parameters for β-quartz. Columns 3 to 5 correspond to equations (17) to (19) of the article. Columns 6, 7 and 8 contain unit cell volumes, SiO_4 tetrahedral volumes and the fractional space occupied by SiO_4-tetrahedra, respectively. Columns 9 to 11 contain tetrahedral distortion parameters as discussed in §3.6 of the article.

T(K)	L(Å)	a_{PC}(Å)	b_{PC}(Å)	c_{PC}(Å)	V_{UC}(Å^3)	V_{tetra}(Å^3)	$3V_{tetra}/V_{UC}$	λ_{PC}	$\delta_{1,PC}\,[=(x_O - x_C)]$	$\delta_{2,PC}\left[=\left(\frac{\sqrt{3}}{9}\frac{c}{a} - x_C\right)\right]$
860	2.49690(5)	1.809(2)	1.860(3)	1.8183(1)	117.811(6)	2.040(1)	0.05194(3)	0.0111(9)	-0.0021(2)	-0.00110(1)
875	2.49785(5)	1.811(2)	1.858(3)	1.8188(1)	117.929(6)	2.041(1)	0.05191(3)	0.0105(9)	-0.0020(2)	-0.00113(1)
890	2.49810(5)	1.811(2)	1.859(3)	1.8189(1)	117.959(6)	2.041(1)	0.05192(3)	0.0107(9)	-0.0020(2)	-0.00114(1)
906	2.49820(5)	1.812(2)	1.858(3)	1.8188(1)	117.967(6)	2.041(1)	0.05190(3)	0.0102(9)	-0.0019(2)	-0.00115(1)
921	2.49830(5)	1.812(2)	1.858(3)	1.8188(1)	117.974(6)	2.041(1)	0.05190(3)	0.0103(9)	-0.0019(2)	-0.00117(1)
937	2.49830(5)	1.812(2)	1.858(3)	1.8187(1)	117.970(6)	2.041(1)	0.05190(3)	0.0103(9)	-0.0019(2)	-0.00117(1)
953	2.49830(5)	1.812(2)	1.858(3)	1.8187(1)	117.967(6)	2.041(1)	0.05190(3)	0.0103(9)	-0.0019(2)	-0.00118(1)
974	2.49830(5)	1.812(2)	1.858(3)	1.8185(1)	117.957(6)	2.041(1)	0.05190(3)	0.0103(9)	-0.0019(2)	-0.00120(1)
987	2.49830(5)	1.814(2)	1.855(3)	1.8184(1)	117.948(6)	2.040(1)	0.05188(3)	0.0096(9)	-0.0018(2)	-0.00121(1)
999	2.49830(5)	1.814(2)	1.855(3)	1.8184(1)	117.946(6)	2.039(1)	0.05187(3)	0.0094(9)	-0.0017(2)	-0.00122(1)
1015	2.49835(5)	1.815(2)	1.853(3)	1.8183(1)	117.946(6)	2.039(1)	0.05185(3)	0.0089(9)	-0.0016(2)	-0.00123(1)

1030	2.49830(5)	1.815(2)	1.853(3)	1.8182(1)	117.937(6)	2.038(1)	0.05185(3)	0.0089(9)	-0.0016(2)	-0.00123(1)
1046	2.49830(5)	1.814(2)	1.855(3)	1.8182(1)	117.933(6)	2.039(1)	0.05188(3)	0.0096(9)	-0.0018(2)	-0.00124(1)
1061	2.49825(5)	1.814(2)	1.854(3)	1.8181(1)	117.926(6)	2.039(1)	0.05186(3)	0.0092(9)	-0.0017(2)	-0.00124(1)
1076	2.49820(5)	1.815(2)	1.853(3)	1.8180(1)	117.910(6)	2.038(1)	0.05185(3)	0.0089(9)	-0.0016(2)	-0.00125(1)
1092	2.49825(5)	1.815(2)	1.852(3)	1.8179(1)	117.913(6)	2.038(1)	0.05184(3)	0.0087(9)	-0.0016(2)	-0.00126(1)
1107	2.49825(5)	1.815(2)	1.853(3)	1.8179(1)	117.911(6)	2.038(1)	0.05185(3)	0.0090(9)	-0.0016(2)	-0.00126(1)
1122	2.49820(5)	1.818(2)	1.847(3)	1.8178(1)	117.897(6)	2.035(1)	0.05178(3)	0.0071(9)	-0.0012(2)	-0.00128(1)
1138	2.49810(5)	1.818(2)	1.848(3)	1.8176(1)	117.879(6)	2.035(1)	0.05179(3)	0.0073(9)	-0.0013(2)	-0.00128(1)
1152	2.49800(5)	1.818(2)	1.848(3)	1.8175(1)	117.859(6)	2.035(1)	0.05179(3)	0.0073(9)	-0.0013(2)	-0.00129(1)
1168	2.49795(5)	1.818(2)	1.847(3)	1.8174(1)	117.848(6)	2.034(1)	0.05178(3)	0.0071(9)	-0.0012(2)	-0.00130(1)
1183	2.49785(5)	1.822(2)	1.839(4)	1.8172(1)	117.830(6)	2.030(2)	0.05170(4)	0.0048(11)	-0.0007(2)	-0.00131(1)
1198	2.49775(5)	1.821(2)	1.841(4)	1.8171(1)	117.812(6)	2.031(2)	0.05171(4)	0.0053(11)	-0.0008(2)	-0.00132(1)
1213	2.49765(5)	1.824(2)	1.836(4)	1.8170(1)	117.796(6)	2.028(2)	0.05165(4)	0.0036(9)	-0.0005(3)	-0.00132(1)
1235	2.49755(10)	1.825(3)	1.835(5)	1.8169(1)	117.778(10)	2.027(2)	0.05164(6)	0.0034(12)	-0.0004(4)	-0.00133(1)

References

Antao, S.M. (2016). *Acta Cryst.* B**72**, 249-262

Hahn, T. (1995). Editor. *International Tables for Crystallography*, Vol. A, *Space-Group Symmetry*. Dordrecht: Kluwer.

Haines, J., Cambon, O., Philippot, E., Chapon, L. & Hull, S. (2002). *J. Solid State Chem.* 166, 434-441

Reifenberg, M. & Thomas, N.W. (2018). *Acta Cryst.* B**74**, 165-181
